# Brain capillary structures of schizophrenia cases and controls show a correlation with their neuron structures

**DOI:** 10.1038/s41598-021-91233-z

**Published:** 2021-06-03

**Authors:** Rino Saiga, Masayuki Uesugi, Akihisa Takeuchi, Kentaro Uesugi, Yoshio Suzuki, Susumu Takekoshi, Chie Inomoto, Naoya Nakamura, Youta Torii, Itaru Kushima, Shuji Iritani, Norio Ozaki, Kenichi Oshima, Masanari Itokawa, Makoto Arai, Ryuta Mizutani

**Affiliations:** 1 Department of Applied Biochemistry, Tokai University, Hiratsuka, Kanagawa 259-1292 Japan; 2 Japan Synchrotron Radiation Research Institute (JASRI/SPring-8), Sayo, Hyogo 679-5198 Japan; 3 Photon Factory, High Energy Accelerator Research Organization KEK, Tsukuba, Ibaraki 305-0801 Japan; 4 Department of Cell Biology, Tokai University School of Medicine, Isehara, Kanagawa 259-1193 Japan; 5 Department of Pathology, Tokai University School of Medicine, Isehara, Kanagawa 259-1193 Japan; 6 Department of Psychiatry, Nagoya University Graduate School of Medicine, Nagoya, Aichi 466-8550 Japan; 7 Medical Genomics Center, Nagoya University Hospital, Nagoya, Aichi 466-8550 Japan; 8 Tokyo Metropolitan Matsuzawa Hospital, Setagaya, Tokyo 156-0057 Japan; 9 Tokyo Metropolitan Institute of Medical Science, Setagaya, Tokyo 156-8506 Japan

**Keywords:** Imaging techniques, X-ray tomography, Schizophrenia

## Abstract

Brain blood vessels constitute a micrometer-scale vascular network responsible for supply of oxygen and nutrition. In this study, we analyzed cerebral tissues of the anterior cingulate cortex and superior temporal gyrus of schizophrenia cases and age/gender-matched controls by using synchrotron radiation microtomography or micro-CT in order to examine the three-dimensional structure of cerebral vessels. Over 1 m of cerebral blood vessels was traced to build Cartesian-coordinate models, which were then used for calculating structural parameters including the diameter and curvature of the vessels. The distribution of vessel outer diameters showed a peak at 7–9 μm, corresponding to the diameter of the capillaries. Mean curvatures of the capillary vessels showed a significant correlation to the mean curvatures of neurites, while the mean capillary diameter was almost constant, independent of the cases. Our previous studies indicated that the neurites of schizophrenia cases are thin and tortuous compared to controls. The curved capillaries with a constant diameter should occupy a nearly constant volume, while neurons suffering from neurite thinning should have reduced volumes, resulting in a volumetric imbalance between the neurons and the vessels. We suggest that the observed structural correlation between neurons and blood vessels is related to neurovascular abnormalities in schizophrenia.

## Introduction

Brain blood vessels constitute a micrometer-scale vascular network that is responsible for supplying oxygen and nutrition to brain cells^1^. The capillary bed of the vascular network contributes to relatively uniform cortical tissue perfusion and oxygenation^2^. It has been reported that capillary density varies depending on the local metabolism of the brain tissue^3^, indicating that local brain activity is related to the capillary network^4^. Cerebral oxygen metabolism and blood flow have been shown to be affected in psychiatric and neurological disorders including schizophrenia^5^, bipolar disorder^6^, Alzheimer's disease^7^ and mitochondrial encephalomyopathy^8^. The coupling between the brain tissue metabolism and the blood flow is maintained by a neurovascular unit organized from brain tissue cells, such as neurons and capillary endothelial cells^9^. Although the capillary diameter of human cerebral tissue has been studied using light and electron microscopy images^10,11^, the structural correlation between the capillary and the neuron has yet to be revealed.

In schizophrenia, the neurovascular coupling between the cerebral glucose metabolism and the cerebral blood flow shows abnormalities compared with controls^12^. Brain regions including the anterior cingulate cortex and the temporal lobe have been shown to differ with respect to their cerebral blood flow in schizophrenia^13,14^. An analysis of cerebral hemoglobin concentrations of schizophrenia patients using near-infrared spectroscopy indicated a significant association of the untreated-psychosis duration to the decreased cortical activity of temporal lobe areas^15^. A volumetric change in brain tissue^16–18^ has been reported for schizophrenia, suggesting that structural changes in brain-tissue components accompany the disorder. It has also been reported that schizophrenia patients exhibit elevated mortality from ischemic heart disease^19^, indicating that schizophrenia is accompanied by lesions in the cardiovascular system. These physiological and epidemiological studies suggest the relevance of blood vessels to schizophrenia etiology. Although genes associated with schizophrenia have been identified from a genome-wide association study^20^, their involvement in the vascular system has not been sufficiently examined and the pathological changes to vessels have not been fully delineated^12^.

We have recently reported nanometer-scale three-dimensional studies on cerebral tissues of schizophrenia and age/gender-matched controls by using synchrotron radiation nanotomography^21,22^. The analyses of the anterior cingulate cortex and superior temporal gyrus revealed that neurites of schizophrenia cases are thin and tortuous while those of controls are thick and straight. Frequency distributions of the neurite curvature showed similar profiles within the same brain area of the same individual, but are significantly dissimilar between brain areas and the dissimilarity varies from case to case. These results indicated that the structures of cerebral neurons vary between individuals and become extraordinary in schizophrenia^22^. We applied these findings to an artificial neural network to examine the functional relevance of the structural change in the computational model. The neurite thinning in schizophrenia was implemented in the artificial neural network by applying restrictions to its connection parameters. The resultant schizophrenia mimicking network outperformed the conventional AI in image recognition tasks^23^. It has been reported that polygenic risk scores of schizophrenia and bipolar disorder are associated with membership in artistic societies and creative professions^24^. This is consistent with the outperformance of the artificial neural network mimicking the neuron structure in schizophrenia. Further analysis of brain tissues should delineate the involvement of other tissue constituents such as blood vessels to the neuropathology of schizophrenia.

In this study, we analyzed blood vessel networks of cerebral tissues of four schizophrenia cases (hereafter called S1–S4; Supplementary Table S1) and of four age/gender-matched control cases (N1–N4; Supplementary Table S1). Post-mortem brain tissues of the Brodmann area 22 (BA22) of the superior temporal gyrus and BA24 of the anterior cingulate cortex were subjected to Golgi staining and then to synchrotron radiation microtomography during 2011–2018 to visualize their three-dimensional structures. The obtained images were traced to build Cartesian-coordinate models of vessel networks, of which the coordinates were used for calculating structural parameters, such as the diameter and curvature of the capillary vessels. The resultant structural parameters were analyzed to examine the structural relationship between capillary vessels and neurons.

## Results

### Structural analysis of vessel network

**Figure 1:**
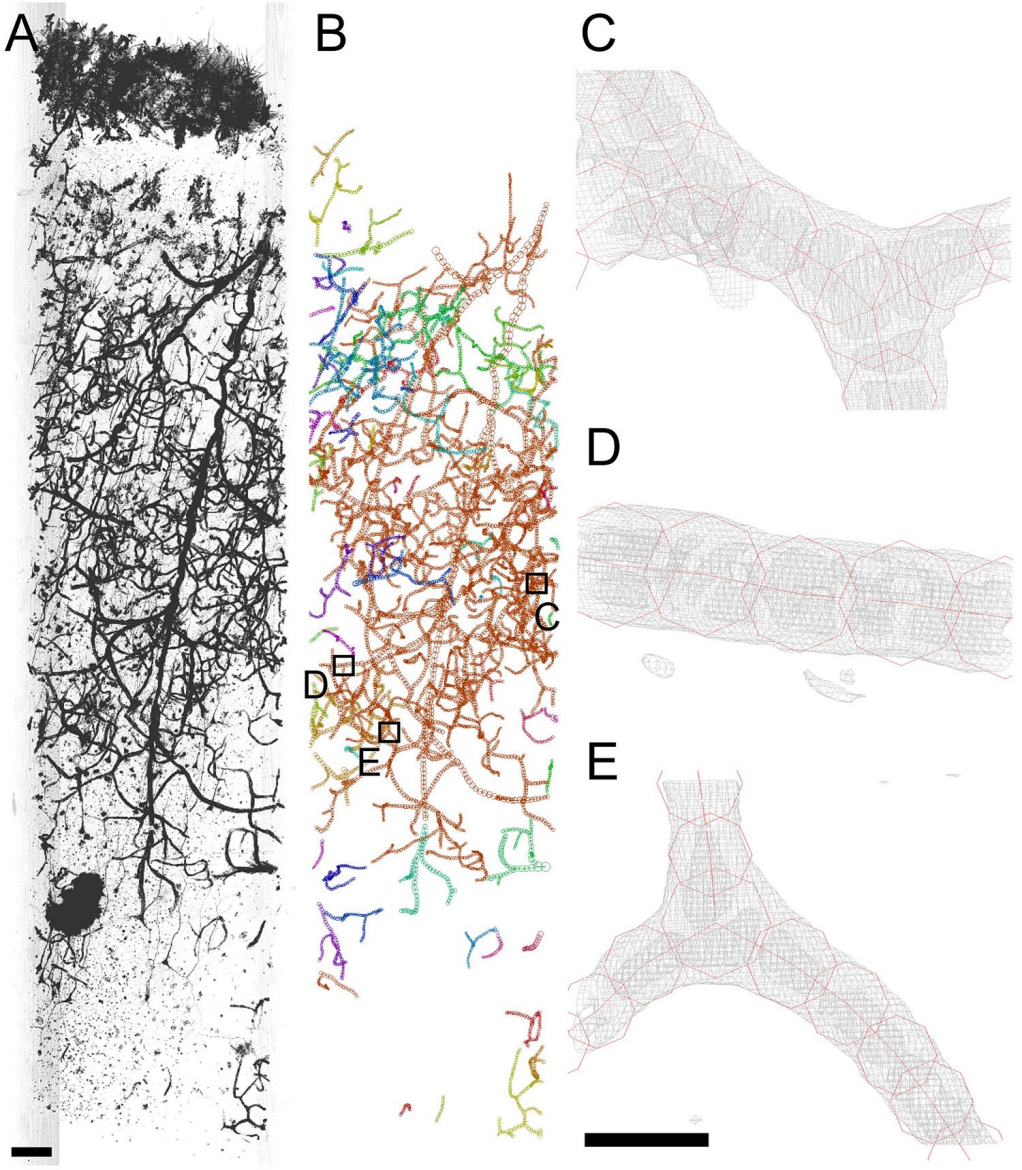
Rendering of three-dimensional image of BA22 cerebral tissue of schizophrenia case S1 and Cartesian-coordinate model of its vessel network. The pial surface is toward the top. (**A**) Three-dimensional image of the tissue. Linear attenuation coefficients of 8–50 cm^−1^ were rendered in gray scale with the maximum projection method of the VG Studio software. Scale bar: 100 μm. (**B**) Cartesian-coordinate model of vessel network built by tracing the three-dimensional image. The model is viewed from nearly the same direction as the rendering. The vessel models of the other 23 samples were built in the same manner (Supplementary Figures S1–S17). The model was drawn with the MCTrace software. Model constituents are color-coded. The vessels magnified in panels **C**–**E** are indicated with boxes. (**C**–**E**) Cartesian-coordinate models of capillary vessels (red) superposed on cage representations of the three-dimensional image (gray) contoured at 6 times the standard deviation of the image from its mean intensity. Blood cells were visualized in the vessel lumen as low-intensity bodies. Positions of these vessels are indicated with boxes in (**B**). Scale bar: 10 μm.

Three-dimensional structures of blood vessel networks in the brain tissues of BA22 of the superior temporal gyrus and BA24 of the anterior cingulate cortex were visualized by using synchrotron radiation microtomography or micro-CT^25^. The tissue images of the schizophrenia S1–S4 and control N1–N4 cases are shown in Fig. 1 and Supplementary Figures S1–S17. Experimental conditions and statistics of the structural reconstruction in Cartesian coordinate space are summarized in Supplementary Tables S1–S3. A total of over 1 m of blood vessels was reconstructed with these procedures (Fig. 1, Supplementary Video S1, Supplementary Figures S1–S17). The spatial resolution of the micro-CT images of this study was determined to be 1.2–1.6 µm (Supplementary Table S2). The precision of the structural parameters should be less than 0.2 µm according to the estimation measure we previously reported^21^.

**Figure 2:**
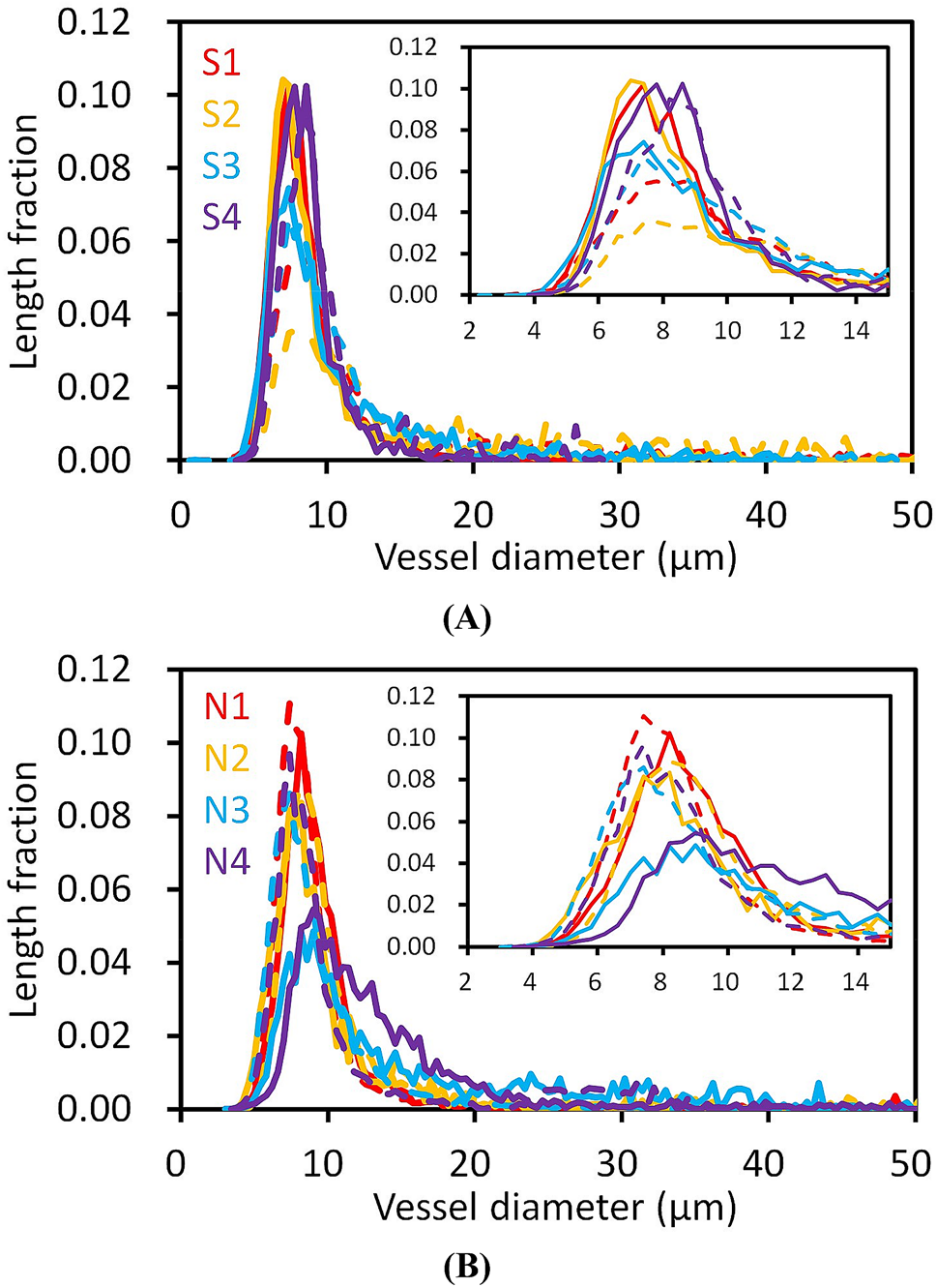
Vessel diameter distribution. The frequency distribution in each 0.4-μm diameter bin is represented by the length fraction, which was calculated by dividing the vessel length per bin by the total vessel length. Insets show magnifications of capillary peaks. Schizophrenia cases S1–S4 and controls N1–N4 are color-coded. Solid lines represent BA22 distributions, and dashed lines represent BA24 distributions. (**A**) Diameter distribution of the schizophrenia cases. (**B**) Diameter distribution of the control cases.

The reconstructed vessel structures were evaluated by calculating structural parameters from their Cartesian coordinates. Figure 2 shows the frequency distribution of vessel diameters in each 0.4-μm diameter bin. The frequency distribution is represented using the length fraction, which was calculated by dividing the vessel length per bin by the total length. All the datasets of BA22 and BA24 of the schizophrenia and control cases showed major peaks at 7.0 µm to 9.0 µm, which correspond to capillary vessels. The vessel diameters evaluated in this study include the thickness of the vessel walls and represent the outer dimeter (Fig. 1C–E). Therefore, the peak values were larger than the inner diameter of the cerebral capillary^10,11^ by an amount of vessel wall thickness, as discussed below. The diameter profiles (Fig. 2) indicated that a substantial fraction of vessels had diameters less than 15 µm. Hence, hereafter we will refer to vessel segments having mean diameters less than 15 μm as capillaries. The plots of the BA22 and BA24 areas of the schizophrenia and control cases showed slightly different profiles, though their peak positions were almost the same, indicating that the capillary diameter was virtually constant between cases and between brain areas.

The capillary vessel structures were further analyzed in terms of their geometric parameters. Three-dimensional curves can be represented with two geometric parameters: curvature and torsion (Supplementary Figure S18). The curvature corresponds to the reciprocal of the radius of a curve; hence, it represents the sharpness of the capillary curve. The torsion is the deviation of the curve from a plane; it represents the right/left handedness of a spiral. The frequency distributions of these geometric parameters of capillary vessels are shown in Supplementary Figures S19 and S20.

**Figure 3:**
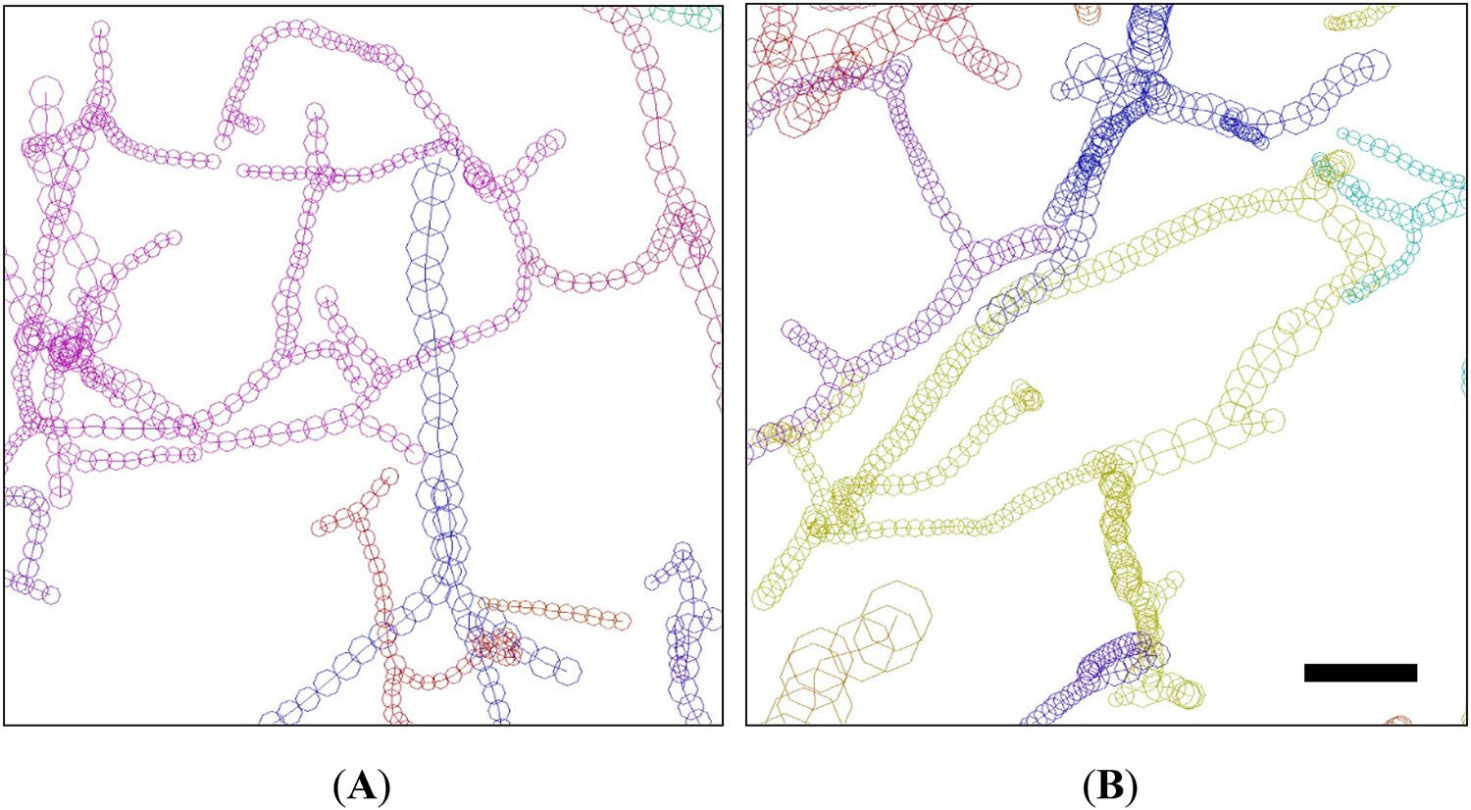
Vessel structures in (**A**) tissue of schizophrenia S3-22 and (**B**) tissue of control N4-22B. The capillary vessels of S3-22 had curved structures, whereas those of N4-22B were rather straight. The pial surface is toward the top. The structures are drawn to the same scale using MCTrace. Nodes composing each vessel are indicated with octagons. The color coding and structural orientation are the same as in Supplementary Figures S2 and S7. Scale bar: 50 μm.

The curvature plots (Supplementary Figure S19) showed single-peak profiles for all of the schizophrenia and control cases. No significant difference in their means was identified between the schizophrenia/control groups or between the brain areas in this study. Figure 3 shows examples of vessel structures showing high and low curvature. The capillary network of S3-22 showed the highest mean curvature of 0.037 µm^−1^ (corresponding to a curve radius of 27 µm), while the capillaries of N4-22B showed the second lowest mean curvature of 0.021 µm^−1^ (corresponding to a curve radius of 48 µm). These parametric differences are discernible in the structures of S3-22 and N4-22B (Fig. 3).

Supplementary Figure S20 shows the torsion distributions. Each torsion profile had a nearly symmetric peak centered at the origin, indicating that the three-dimensional curves of capillary vessels have no handedness. Means and deviations of the capillary torsion showed no significant difference between the schizophrenia/control groups or between the brain areas.

### Structural correlation between capillary vessels and neurons

We previously reported that neurite curvature is significantly higher in schizophrenia cases than in controls^21,22^. The schizophrenia and control cases analyzed in this study were the same as those analyzed in our previous studies on neuron structure. This allowed us to examine correlations between the vessel and neuron structures.

**Figure 4:**
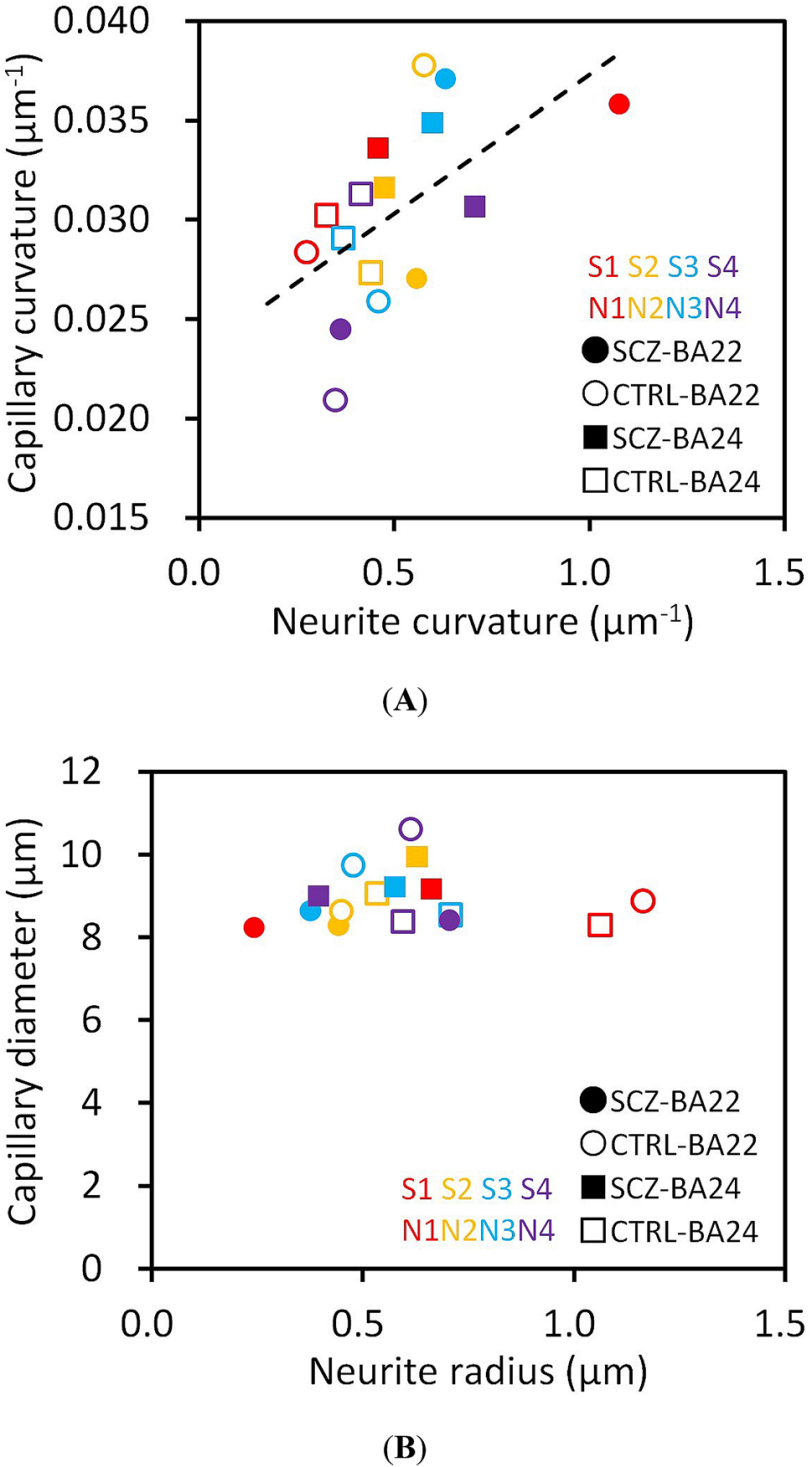
(**A**) Relationship between mean capillary curvature and mean neurite curvature of the BA22 or BA24 area of each case. The dashed line indicates a linear regression. Schizophrenia cases S1–S4 and controls N1–N4 are color-coded. (**B**) Relationship between mean capillary diameter and mean neurite thickness radius. Cases are color-coded as in (**A**).

Figure 4A shows the relationship between the mean capillary curvature and the mean neurite curvature of each brain area of the schizophrenia and control cases. The plot illustrates a significant correlation of the mean curvature of capillary vessels to that of neurites (Spearman's *ρ* = 0.63, *p* = 0.011, *n* = 16), indicating that the brain tissues with tortuous neuronal networks have tortuous capillary vessels. No significant difference in the slope was observed between the schizophrenia and control groups. The mean capillary curvature showed a difference in its variance between brain areas. The capillary curvatures of BA22 were widely distributed, while those of BA24 were limited to a confined range (Fig. 4A), resulting in a significant difference in variance (*p* = 0.019, Bartlett’s test, *n* = 8). We also examined the relation between the capillary diameter and neurite thickness radius (Fig. 4B). In contrast to the curvature correlation, the mean capillary diameter showed no correlation with the neurite thickness radius, but was rather constant regardless of neurite thickness. This result indicates that the capillary vessel size is determined independently of the neuron structure.

## Discussion

The microtomographic visualization of cerebral tissues of the anterior cingulate cortex and superior temporal gyrus of the schizophrenia and control cases and the subsequent analyses by tracing blood vessels in three-dimensional coordinate space revealed structural characteristics of vessel networks in human cerebral tissue. The vessel diameter distribution showed a peak at 7.0–9.0 µm, which represents the capillary outer diameter. The inner diameter of the capillary lumen of human cerebral tissues perfused with acrylic resin was determined to be 7 or 7.5 µm from electron microscopy of resin casts^10^. The mean diameter of the capillary lumen of human cerebral tissue perfused with India ink was determined to be 5.9 or 6.5 µm from confocal images^11^. These two studies reported almost the same diameter, while their procedures were completely different. The capillary outer diameter that was determined without artificial perfusion of any materials in this study (7.0–9.0 µm) is consistent with those inner diameters but thicker by 1–2 µm. This difference is ascribable to the thicknesses of the capillary walls that were included in the diameter evaluation using the Golgi-stained tissues (Fig. 1C–E).

The capillary diameter was almost constant, independent of the neurite diameter (Fig. 4B). This should reflect the physical prerequisite that the capillary must keep its luminal space sufficiently large to allow blood cells to pass through the vessel network. In contrast, the capillary curvature showed a significant correlation to the neurite curvature (Fig. 4A), suggesting a certain structural relationship between the blood vessels and the neurons. A volumetric reduction in cerebral tissue has been reported for schizophrenia^16–18,26–28^. A possible mechanism of this reduction is the reduced neuropil hypothesis^29^, which ascribes the volume change to the neuropil reduction. This is consistent with the neurite thinning observed in our previous studies^21,22^. Neurite thinning reduces the tissue volume, whereas the capillary diameter was found to be constant in this study (Fig. 4B). This results in a shrinkage of the brain tissue, while the sizes of the capillaries remain unchanged, leading to tortuous structures. We suggest that the structure of the vessel network is affected by volumetric changes in cerebral tissue due to neurite thinning.

The cerebral blood flow is coupled to the cerebral metabolism through the neurovascular unit^9^. It has been indicated that the relationship between the cerebral blood flow and the cerebral metabolism in schizophrenia shows abnormalities depending on the area in the brain^12^. This study revealed that capillary curvature correlates with neurite curvature (Fig. 4A), while mean capillary diameter remains constant (Fig. 4B). The constant-diameter capillary in the neurite-thinned tissue of the schizophrenia cases^21,22^ should occupy a larger volumetric fraction compared with those of the controls. If the number of vessel segments in the volume is the same, this density imbalance between the blood vessel and the neuron may lead to an altered relationship between cerebral blood flow and the cerebral metabolism, resulting in abnormalities in the neurovascular coupling.

A cohort study revealed elevated mortality of schizophrenia patients due to ischemic heart disease alongside cancer^19^. The relevance of cerebral microvasculature to schizophrenia has been suggested in genetic and postmortem gene expression studies^30^. A functional genomic study of schizophrenia-associated genes indicated a significant overrepresentation of genes involved in vascular function, vasoregulation and cerebral ischemia^31^. It has been reported that schizophrenia cases show high Bax/Bcl-2 ratio without caspase-3 activation in the temporal cortex^32^. A study on genome-wide mRNA expression in the BA22 area revealed that tissue remodeling, wound repair, and apoptosis showed the strongest process enrichment^33^, which should be related to the possible ischemia. These findings on the involvement of the vascular system in schizophrenia is consistent with the structural correlation between neurons and blood vessels (Fig. 4A) along with neurite thinning in schizophrenia^21,22^.

The mean capillary curvature of BA22 of each case showed a significantly wider distribution than that of BA24 (Fig. 4A). We previously reported a significant difference in spine length between the BA22 and BA24 areas^22^. These areal differences in cerebral tissue structures should reflect functional differences and transcriptional variation between brain areas^34^.

The x-ray microtomographic visualization of the Golgi-stained brain tissues illustrated three-dimensional vessel structures including blood cells inside the capillary lumen (Fig. 1C–E). Light microscopy cannot visualize objects behind stained structures and shows blurring depending on the tissue depth. This should result in systematic biases in the structural analysis. X-ray microtomography passes x-rays through a biological tissue sample that is being rotated; hence, it can uniformly visualize objects even behind stained structures. The resultant three-dimensional image and its reconstruction in Cartesian coordinate space allowed us to evaluate the structures quantitatively using geometric parameters, such as the vessel curvature. A major limitation of this study is the size of the group subjected to the structural analysis. Although a significant correlation between the capillary curvature and the neurite curvature was identified from the analysis of over 1 m of vessels in two brain areas of four schizophrenia and four control cases (Fig. 4A), the number of cases was limited by the availability of beamtime at the synchrotron radiation facility. Another limitation of this study is the sporadic or random property of the Golgi staining that visualizes only parts of the entire vessel network. The present results should, therefore, be regarded as representing a subset of the entire vessel network of the analyzed samples.

Although post-mortem studies on brain tissues of schizophrenia cases revealed ultrastructural damage relevant to capillaries^35^ and elevated fibrin accumulation in the vascular endothelium^36^, the pathology of cerebral vessels in the post-mortem brain has not been fully understood for schizophrenia^12^. This study revealed a correlation of the capillary curvature to the neurite curvature (Fig. 4A). Our previous studies^21,22^ illustrated that the neurite curvature is significantly higher in schizophrenia cases compared with controls. Further studies on the blood vessel networks of the human cerebral tissue should be undertaken to delineate the underlying mechanism of neurovascular abnormalities in schizophrenia.

## Materials and methods

### Cerebral tissue samples

All post-mortem human cerebral tissues were collected with informed consent from the legal next of kin using protocols approved by the Clinical Study Reviewing Board of Tokai University School of Medicine (application no. 07R-018) and the Ethics Committee of Tokyo Metropolitan Institute of Medical Science (approval no. 17–18). This study was conducted according to the Declaration of Helsinki under the approval of the Ethics Committee for the Human Subject Study of Tokai University (approval nos. 11060, 11114, 12114, 13105, 14128, 15129, 16157, 18012, 19001, 20021, and 20022). The schizophrenia patients S1–S4 and control cases N1–N4 (Supplementary Table S1) of this study are the same as those analyzed in our previous report on neuron structure^21,22^. Cerebral tissues of Brodmann area 22 (BA22) of the superior temporal gyrus and BA24 of the anterior cingulate cortex were collected from the left hemispheres of the post-mortem brains and subjected to Golgi impregnation^37^. As indicated in our previous studies^21,22,37,38^, the Golgi protocol used in this study mainly stains neurons and blood vessels. The Golgi-stained tissues were then embedded in borosilicate glass capillaries using epoxy resin, as described previously^21^.

### Microtomography

Tissue structures were visualized with simple projection microtomography at the BL20XU beamline^25^ of SPring-8 from 2011 to 2018, using monochromatic radiation at 12 keV. Absorption contrast images of the brain tissues were recorded with CMOS-based imaging detectors (ORCA-Flash, Hamamatsu Photonics, Japan). The experimental conditions are summarized in Supplementary Table S2. Photon flux at the sample position was estimated to be 3.8 × 10^11^ photons/mm^2^/s during the 2018 beamtime by using Al_2_O_3_:C dosimeters (Nagase-Landauer, Japan), as reported previously^21^. Spatial resolution was estimated to be 1.2–1.6 µm for each beamtime by using three-dimensional test patterns^39^ and from the Fourier domain plot^40^. The tissue samples used for the microtomographic visualization are the same as those used in our previous studies on neuron structure^21,22^, except for samples of N1-22B, N2-24A, and N4-22B (Supplementary Table S3).

### Structural analysis

Tomographic slices were reconstructed with the convolution-back-projection method using the RecView software^38^, as reported previously^21^. The reconstruction calculation was performed by RS. The obtained datasets of three-dimensional tissue images were subjected to manual tracing to build Cartesian-coordinate models of the vessel network. The vessel tracing was performed by using the in-house MCTrace software^41^. The first round of the tracing was conducted from 2012 to 2019 using 10 datasets of S1-24 (BA24 of case S1), S3-24A, S3-24B, S3-24C, S4-24, N1-24A, N1-24B, N2-24B, N3-24A, and N4-24 (Supplementary Table S3). The vessel tracing for these initial datasets were performed without blinding in order to establish the reconstruction procedure and to examine its feasibility. Blood vessel structures were traced by placing spherical nodes and by connecting those nodes to build a vessel model composed of cylindrical segments so as to reproduce the three-dimensional image of the vessel in the Cartesian coordinate space. The vessel model of the S1-24 dataset was built by RM, those of N1-24B and N3-24A by RM and RS, and the other initial datasets by RS. The obtained results were used to verify the methodological feasibility and to improve the model building procedure. The final working models built using these datasets indicated that Cartesian-coordinate models of the blood vessel network can be reconstructed with this method, as shown in Supplementary Figures S1–S17. Finally, all the structures of the initial datasets were examined and edited by RM in 2020 to standardize the tracing results.

The other 14 datasets were subjected to the same reconstruction procedure in 2020 with the role allotment of data management/blinding to RS and data analysis to RM. RS coded data names in order to eliminate human biases in the model building procedure. The datasets was provided to RM in two batches without the case number or the brain area information. Each dataset was divided into slabs consisting of typically 500 tomographic slices in order to accommodate the entire slab in the memory of the PCs used for the structural analysis. Each slab was first examined by displaying the three-dimensional image with 3× binning so that the entire structure was viewed to place an initial node, from which the vessel tracing was started. Then, the three-dimensional image was displayed without binning to precisely trace blood vessels in Cartesian coordinate space. The display threshold was set to 3 to 5 times the noise intensity to keep the tracing criteria constant. When the vessel structure was traced to the end of its image, another starting node was searched for in the 3×-binned map again and subjected to the same procedure. After the vessel tracing was completed for the working image slab, the next slab was loaded and the same process was repeated to reconstruct the whole vessel network.

The first round of the blinded model-building was conducted on 11 datasets designated later as S1-22, S2-22, S2-24A, S2-24B, S3-22, S4-22, N1-22A, N2-22, N3-22, N3-24B, and N4-22A (Supplementary Table S3). The obtained models were locked down and re-assigned to individual cases to aggregate vessel lengths from the 11 datasets along with the initial 10 datasets that were built without blinding as described above. Since the aggregated vessel lengths of BA22 of case N1, BA24 of N2, and BA22 of N4 were less than 10 mm, three additional datasets (which were later designated as N1-22B, N2-24A, and N4-22B; Supplementary Table S3) were chosen from the data stock and provided to RM as the second batch without the case number or brain area information. Vessel networks of the second batch datasets were built with the same procedure. After the models for the three additional datasets were completed, they were locked down and re-assigned to the cases.

Finally, the obtained Cartesian-coordinate models of the vessel networks of the 24 datasets were subjected to automatic geometric analysis using the MCTrace software, as reported previously^21^. The vessel length was calculated by summing the lengths of the edges connecting the vessel node centers. Curvature and torsion of vessels were calculated from the edge trajectory. Mean values of diameter, curvature, and torsion were calculated by weighting them with the vessel length. The statistics of the obtained structures are summarized in Supplementary Table S3.

The RecView software and its source code used for the tomographic reconstruction are available from https://mizutanilab.github.io under the BSD 2-Clause License. The model building and geometric analysis procedures were implemented in the MCTrace software available from the same site under the BSD 2-Clause License.

### Statistical tests

Statistical tests of structural parameters were performed using the R software. Significance was defined as *p* < 0.05. The correlation between the curvatures of capillary vessels and the neurites was examined using Spearman's rank correlation coefficient. The difference in variance of mean capillary curvature was examined using Bartlett's test. The standard error of the weighted mean was calculated using the ratio variance approximation^42,43^.

## Supplementary Information

Supplementary Information 1.

Supplementary Information 2.

Supplementary Video S1.

## References

[1] Cipolla, M. J. Chapter 2 Anatomy and Ultrastructure. In *The cerebral circulation*, 3–14 (Morgan & Claypool Life Sciences, 2009). 21452434

[2] Gould IG, Tsai P, Kleinfeld D, Linninger A (2017). The capillary bed offers the largest hemodynamic resistance to the cortical blood supply. J. Cereb. Blood Flow Metab..

[3] Klein B, Kuschinsky W, Schröck H, Vetterlein F (1986). Interdependency of local capillary density, blood flow, and metabolism in rat brains. Am. J. Physiol..

[4] Xu K, Lamanna JC (2006). Chronic hypoxia and the cerebral circulation. J. Appl. Physiol..

[5] Liu F, Zhuo C, Yu C (2016). Altered cerebral blood flow covariance network in schizophrenia. Front. Neurosci..

[6] Karthikeyan S, Fiksenbaum L, Grigorian A, Lu H, MacIntosh BJ, Goldstein BI (2019). Normal cerebral oxygen consumption despite elevated cerebral blood flow in adolescents with bipolar disorder: Putative neuroimaging evidence of anomalous energy metabolism. Front. Psychiatry.

[7] Ishii K, Kitagaki H, Kono M, Mori E (1996). Decreased medial temporal oxygen metabolism in Alzheimer's disease shown by PET. J. Nucl. Med..

[8] Shishido F, Uemura K, Inugami A, Tomura N, Higano S, Fujita H (1996). Cerebral oxygen and glucose metabolism and blood flow in mitochondrial encephalomyopathy: A PET study. Neuroradiology.

[9] Muoio V, Persson PB, Sendeski MM (2014). The neurovascular unit—concept review. Acta Physiol..

[10] Reina-De La Torre F, Rodriguez-Baeza A, Sahuquillo-Barris J (1998). Morphological characteristics and distribution pattern of the arterial vessels in human cerebral cortex: A scanning electron microscope study. Anat. Rec..

[11] Cassot F, Lauwers F, Fouard C, Prohaska S, Lauwers-Cances V (2006). A novel three-dimensional computer-assisted method for a quantitative study of microvascular networks of the human cerebral cortex. Microcirculation.

[12] Sukumar N, Sabesan P, Anazodo U, Palaniyappan L (2020). Neurovascular uncoupling in schizophrenia: A bimodal meta-analysis of brain perfusion and glucose metabolism. Front. Psychiatry.

[13] Catafau AM, Parellada E, Lomeña FJ, Bernardo M, Pavía J, Ros D (1994). Prefrontal and temporal blood flow in schizophrenia: Resting and activation technetium-99m-HMPAO SPECT patterns in young neuroleptic-naive patients with acute disease. J. Nucl. Med..

[14] Oliveira ÍAF, Guimarães TM, Souza RM, Dos Santos AC, Machado-de-Sousa JP, Hallak JEC (2018). Brain functional and perfusional alterations in schizophrenia: An arterial spin labeling study. Psychiatry Res. Neuroimaging.

[15] Chou P-H, Koike S, Nishimura Y, Kawasaki S, Satomura Y, Kinoshita A (2014). Distinct effects of duration of untreated psychosis on brain cortical activities in different treatment phases of schizophrenia: A multi-channel near-infrared spectroscopy study. Prog. Neuropsychopharmacol. Biol. Psychiatry.

[16] Shenton ME, Dickey CC, Frumin M, McCarley RW (2001). A review of MRI findings in schizophrenia. Schizophr. Res..

[17] Kaur A, Basavanagowda DM, Rathod B, Mishra N, Fuad S, Nosher S (2020). Structural and functional alterations of the temporal lobe in schizophrenia: A literature review. Cureus.

[18] Tang J, Liao Y, Zhou B, Tan C, Liu W, Wang D (2012). Decrease in temporal gyrus gray matter volume in first-episode, early onset schizophrenia: An MRI study. PLoS ONE.

[19] Crump C, Winkleby MA, Sundquist K, Sundquist J (2013). Comorbidities and mortality in persons with schizophrenia: A Swedish national cohort study. Am. J. Psychiatry.

[20] Schizophrenia Working Group of the Psychiatric Genomics Consortium (2014). Biological insights from 108 schizophrenia-associated genetic loci. Nature.

[21] Mizutani R, Saiga R, Takeuchi A, Uesugi K, Terada Y, Suzuki Y (2019). Three-dimensional alteration of neurites in schizophrenia. Transl. Psychiatry.

[22] Mizutani R, Saiga R, Yamamoto Y, Uesugi M, Takeuchi A, Uesugi K (2021). Structural diverseness of neurons between brain areas and between cases. Transl. Psychiatry.

[23] Mizutani, R., Noguchi, S., Saiga, R., Miyashita, M., Arai, M. & Itokawa, M. Schizophrenia-mimicking layers outperform conventional neural network layers. Preprint at https://arxiv.org/abs/2009.10887 (2020). 10.3389/fnbot.2022.851471 PMC8995800 35418846

[24] Power RA, Steinberg S, Bjornsdottir G, Rietveld CA, Abdellaoui A, Nivard MM (2015). Polygenic risk scores for schizophrenia and bipolar disorder predict creativity. Nat. Neurosci..

[25] Suzuki Y, Uesugi K, Takimoto N, Fukui T, Aoyama K, Takeuchi A (2004). Construction and commissioning of a 248 m-long beamline with X-ray undulator light source. AIP Conf. Proc..

[26] Wright IC, Rabe-Hesketh S, Woodruff PW, David AS, Murray RM, Bullmore ET (2000). Meta-analysis of regional brain volumes in schizophrenia. Am. J. Psychiatry.

[27] Olabi B, Ellison-Wright I, McIntosh AM, Wood SJ, Bullmore E, Lawrie SM (2011). Are there progressive brain changes in schizophrenia? A meta-analysis of structural magnetic resonance imaging studies. Biol. Psychiatry.

[28] Haijma SV, van Haren N, Cahn W, Koolschijn PCMP, Pol HEH, Kahn RS (2013). Brain volumes in schizophrenia: A meta-analysis in over 18,000 subjects. Schizophr. Bull..

[29] Selemon LD, Goldman-Rakic PS (1999). The reduced neuropil hypothesis: A circuit based model of schizophrenia. Biol. Psychiatry.

[30] Katsel P, Roussos P, Pletnikov M, Haroutunian V (2017). Microvascular anomaly conditions in psychiatric disease Schizophrenia—angiogenesis connection. Neurosci. Biobehav. Rev..

[31] Moises HW, Wollschläger D, Binder H (2015). Functional genomics indicate that schizophrenia may be an adult vascular-ischemic disorder. Transl. Psychiatry.

[32] Jarskog LF, Selinger ES, Lieberman JA, Gilmore JH (2004). Apoptotic proteins in the temporal cortex in schizophrenia: High Bax/Bcl-2 ratio without caspase-3 activation. Am. J. Psychiatry.

[33] Barnes MR, Huxley-Jones J, Maycox PR, Lennon M, Thornber A, Kelly F (2011). Transcription and pathway analysis of the superior temporal cortex and anterior prefrontal cortex in schizophrenia. J. Neurosci. Res..

[34] Hawrylycz MJ, Lein ES, Guillozet-Bongaarts AL, Shen EH, Ng L, Miller JA (2012). An anatomically comprehensive atlas of the adult human brain transcriptome. Nature.

[35] Uranova NA, Zimina IS, Vikhreva OV, Krukov NO, Rachmanova VI, Orlovskaya DD (2010). Ultrastructural damage of capillaries in the neocortex in schizophrenia. World J. Biol. Psychiatry.

[36] Hirai S, Miwa H, Tanaka T, Toriumi K, Kunii Y, Sakamoto T (2020). Brain angiopathy and impaired glucose logistics in mice with psychosis-related higher brain dysfunction. bioRxiv.

[37] Mizutani R, Takeuchi A, Uesugi K, Ohyama M, Takekoshi S, Osamura RY (2008). Three-dimensional microtomographic imaging of human brain cortex. Brain Res..

[38] Mizutani R, Takeuchi A, Uesugi K, Takekoshi S, Osamura RY, Suzuki Y (2010). Microtomographic analysis of neuronal circuits of human brain. Cereb. Cortex.

[39] Mizutani R, Takeuchi A, Uesugi K, Suzuki Y (2008). Evaluation of the improved three-dimensional resolution of a synchrotron radiation computed tomograph using a micro-fabricated test pattern. J. Synchrotron Radiat..

[40] Mizutani R, Saiga R, Takekoshi S, Inomoto C, Nakamura N, Itokawa M (2016). A method for estimating spatial resolution of real image in the Fourier domain. J. Microsc..

[41] Mizutani R, Saiga R, Takeuchi A, Uesugi K, Suzuki Y (2013). Three-dimensional network of Drosophila brain hemisphere. J. Struct. Biol..

[42] Cochran, W.G. Chapter 6, Ratio estimators. In *Sampling Techniques*, 3rd ed, 150–186 (Wiley, 1977).

[43] Gatz DF, Smith L (1995). The standard error of a weighted mean concentration–I. Bootstrapping vs other methods. Atmos. Environ..

