# Supplementary material for: Brain capillary structures of schizophrenia cases and controls show a correlation with their neuron structures
Source: Sci Rep. 2021 Jun 3;11:11768. doi: 10.1038/s41598-021-91233-z (PMC8175464; doi:10.1038/s41598-021-91233-z)
Supplement: Supplementary file 1 — Supplementary Information 1. [file 41598_2021_91233_MOESM1_ESM.pdf]

Supplementary Information of:

Brain capillary structures of schizophrenia cases and controls show a correlation with their neuron structures

Rino Saiga[1], Masayuki Uesugi[2], Akihisa Takeuchi[2], Kentaro Uesugi[2], Yoshio Suzuki[3], Susumu Takekoshi[4], Chie Inomoto[5], Naoya Nakamura[5], Youta Torii[6], Itaru Kushima[6,7], Shuji Iritani[6,8], Norio Ozaki[6,7], Kenichi Oshima[8,9], Masanari Itokawa[8,9], Makoto Arai[9], and Ryuta Mizutani[1,]*

[1]Department of Applied Biochemistry, Tokai University, Hiratsuka, Kanagawa 259-1292, Japan; [2]Japan Synchrotron Radiation Research Institute (JASRI/SPring-8), Sayo, Hyogo 679-5198, Japan; [3]Photon Factory, High Energy Accelerator Research Organization KEK, Tsukuba, Ibaraki 305-0801, Japan; [4]Department of Cell Biology, Tokai University School of Medicine, Isehara, Kanagawa 259-1193, Japan; [5]Department of Pathology, Tokai University School of Medicine, Isehara, Kanagawa 259-1193, Japan; [6]Department of Psychiatry, Nagoya University Graduate School of Medicine, Nagoya, Aichi 466-8550, Japan; [7]Medical Genomics Center, Nagoya University Hospital, Nagoya 466-8550, Aichi, Japan; [8]Tokyo Metropolitan Matsuzawa Hospital, Setagaya, Tokyo 156-0057, Japan; [9]Tokyo Metropolitan Institute of Medical Science, Setagaya, Tokyo 156-8506, Japan
*Correspondence: mizutanilaboratory@gmail.com

Index

Supplementary Figure S1–S7 ... p. 2–8
Supplementary Figure S8–S17 ... p. 9–18
Supplementary Figure S18 ... p. 19
Supplementary Figure S19 ... p. 20
Supplementary Figure S20 ... p. 21
Supplementary Video S1 caption ... p. 22

Supplementary Tables S1–S3 are provided separately.

A B

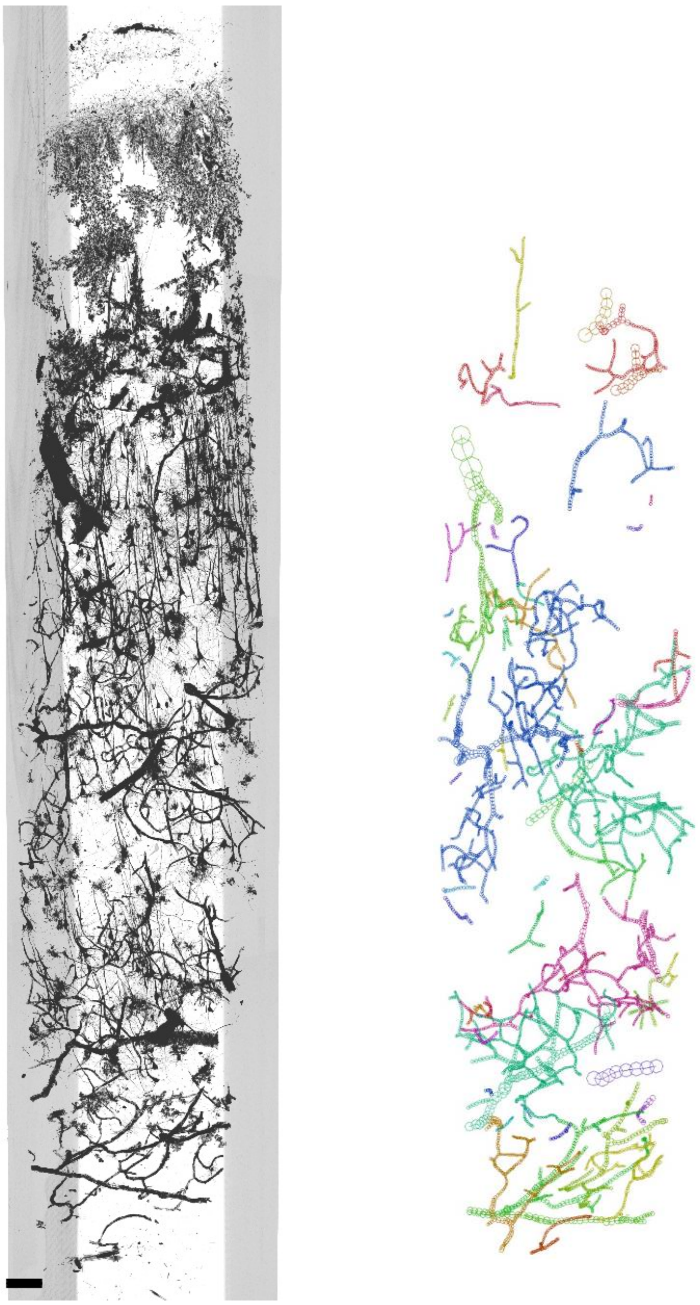

Supplementary Figure S1. Rendering of three-dimensional images of BA22 cerebral tissues and Cartesian-coordinate models of the vessel structures. Renderings and models are viewed from nearly the same direction. The pial surface is toward the top. Images were rendered with the maximum projection method of the VG Studio software. Models were drawn with the MCTrace software. Model constituents are color-coded. Nodes composing vessels are indicated with circles. Scale bars: 100 µm. (**A**) Rendering of image dataset S2-22 of the schizophrenia S2 case. Linear attenuation coefficients of 8–50 cm^{-1} were rendered in gray scale. (**B**) Cartesian-coordinate model of S2-22.

A B

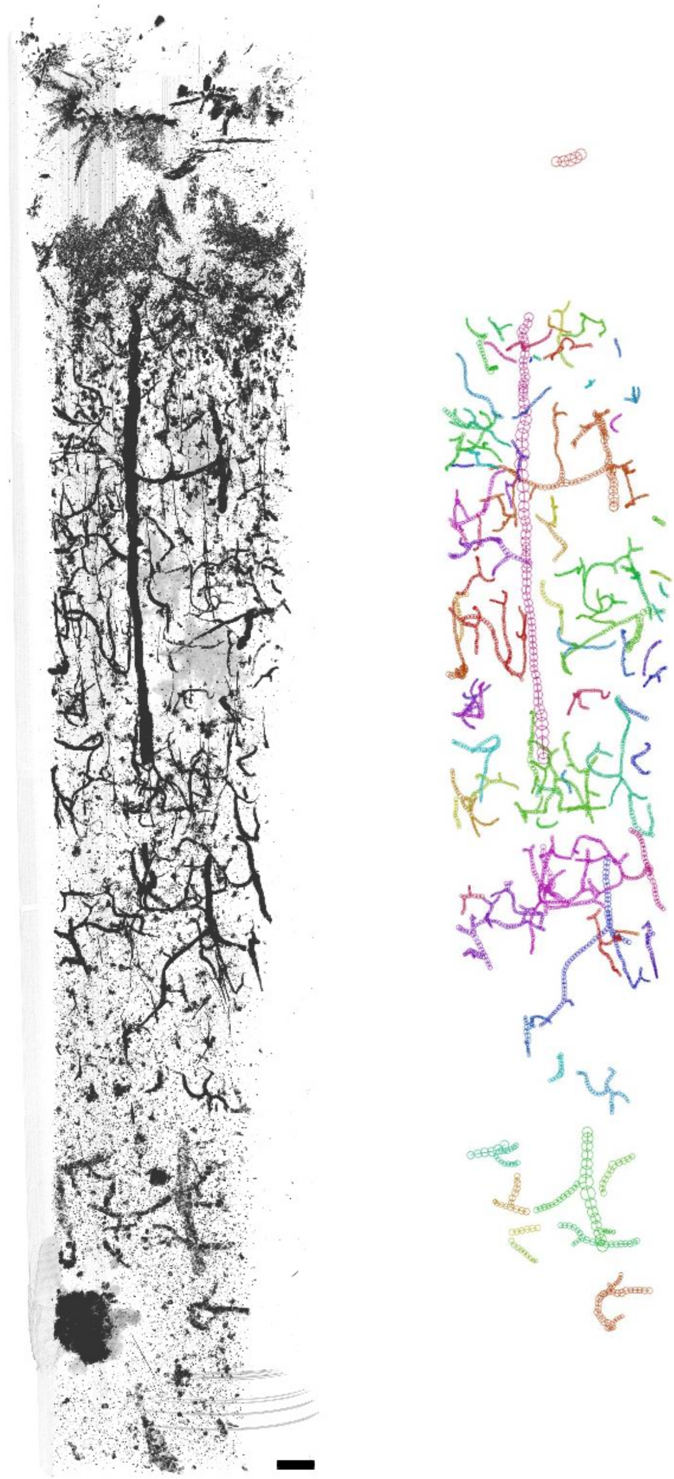

Supplementary Figure S2. Rendering of three-dimensional images of BA22 cerebral tissues and Cartesian-coordinate models of the vessel structures. Scale bars: 100 μm. (**A**) Rendering of schizophrenia dataset S3-22. Linear attenuation coefficients of 8–50 cm^{-1} were rendered in gray scale. (**B**) Model of S3-22.

A B

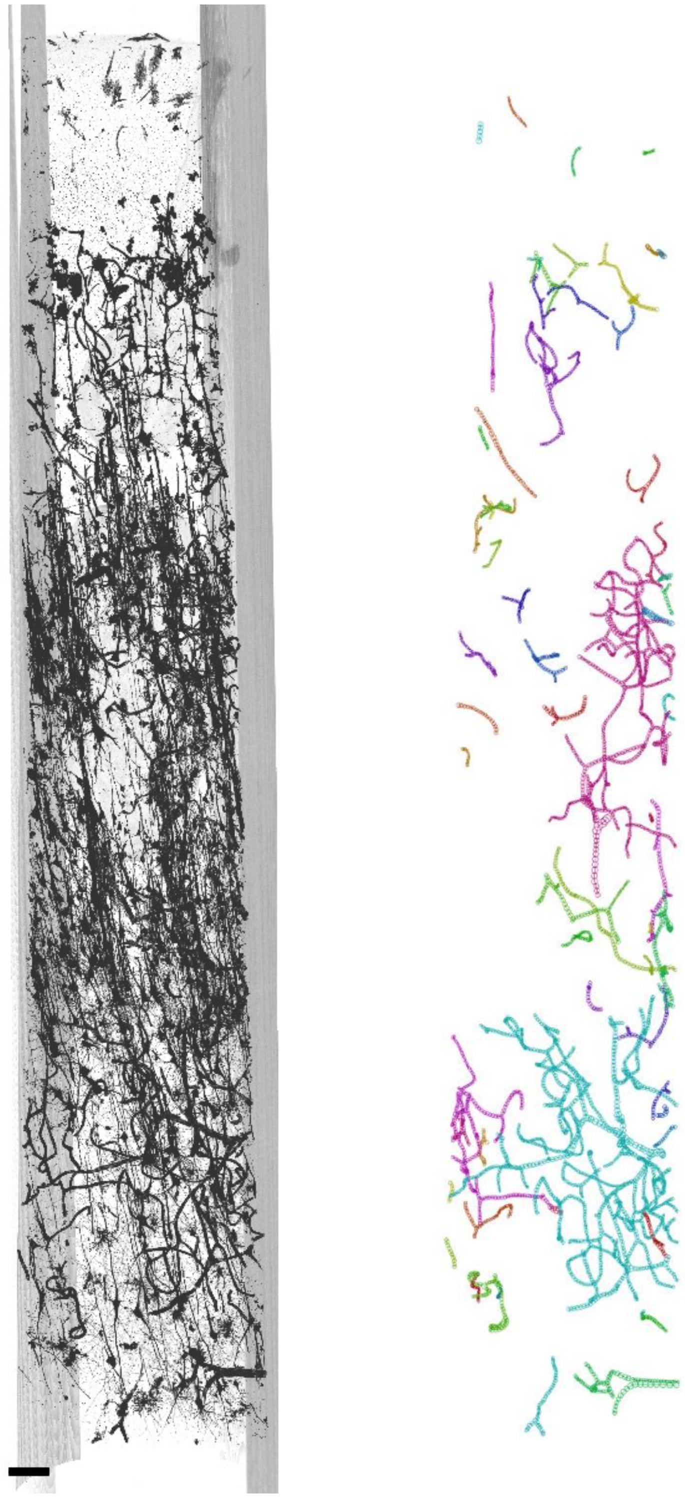

Supplementary Figure S3. Rendering of three-dimensional images of BA22 cerebral tissues and Cartesian-coordinate models of the vessel structures. Scale bars: 100 μm. (**A**) Rendering of schizophrenia dataset S4-22. Linear attenuation coefficients of 8–50 cm^{-1} were rendered in gray scale. (**B**) Model of S4-22.

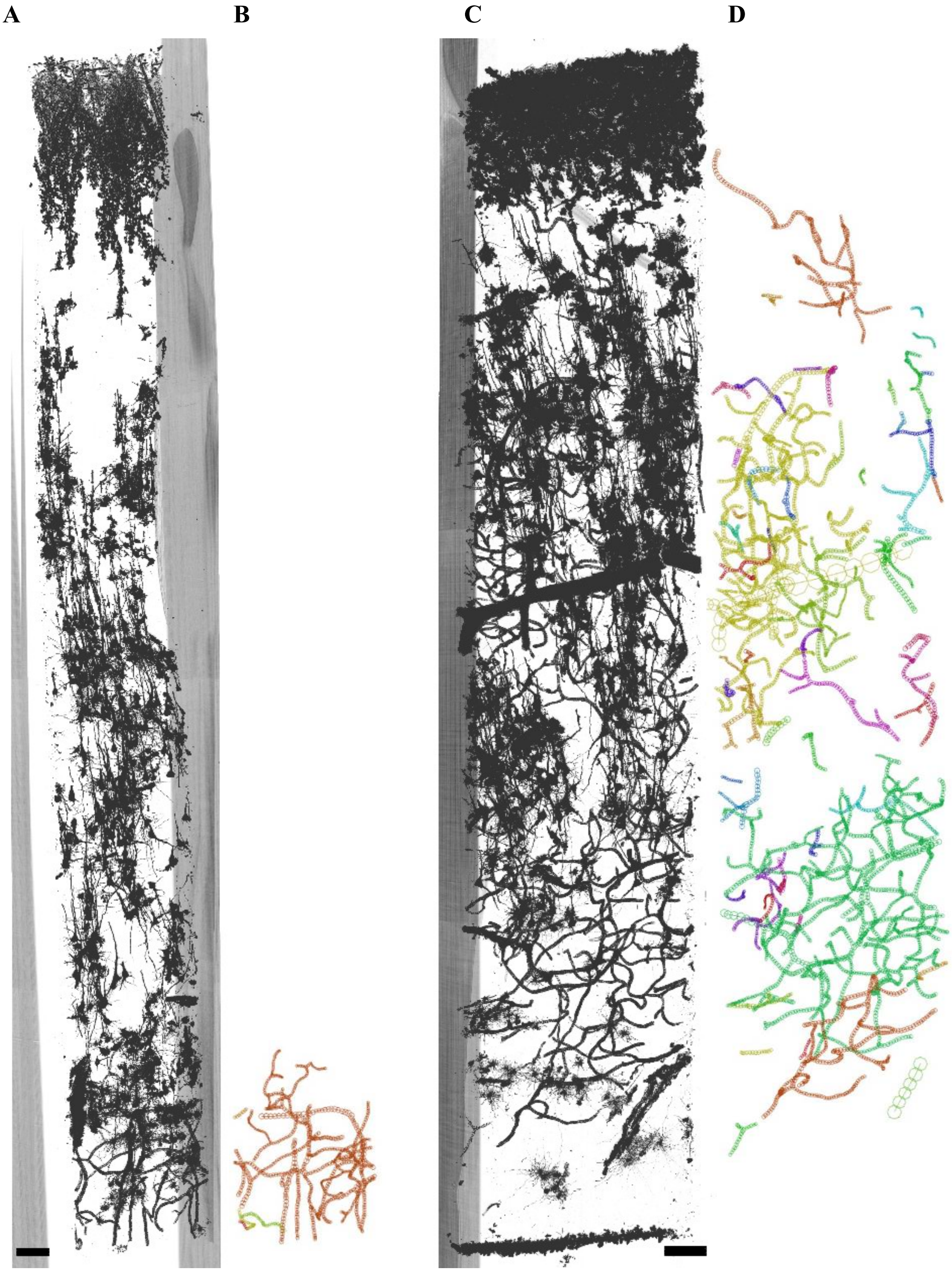

Supplementary Figure S4. Rendering of three-dimensional images of BA22 cerebral tissues and Cartesian-coordinate models of the vessel structures. Scale bars: 100 μm. (**A**) Rendering of control dataset N1-22A. Linear attenuation coefficients of 8–50 cm^{-1} were rendered in gray scale. (**B**) Model of N1-22A. (**C**) Rendering of control dataset N1-22B. Linear attenuation coefficients of 8–50 cm^{-1} were rendered in gray scale. (**D**) Model of N1-22B.

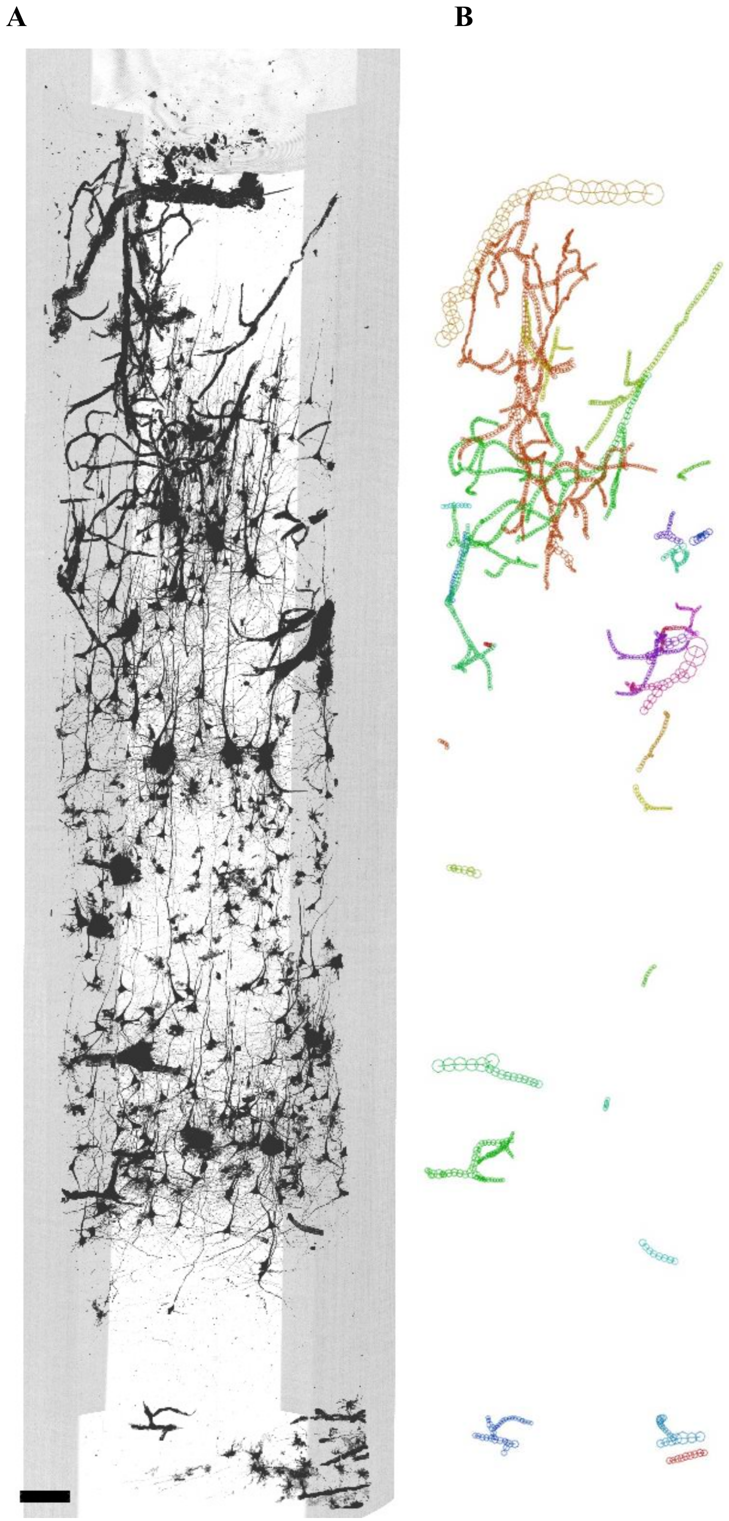

Supplementary Figure S5. Rendering of three-dimensional images of BA22 cerebral tissues and Cartesian-coordinate models of the vessel structures. Scale bars: 100 μm. (**A**) Rendering of control dataset N2-22. Linear attenuation coefficients of 8–50 cm^{-1} were rendered in gray scale. (**B**) Model of N2-22.

A B

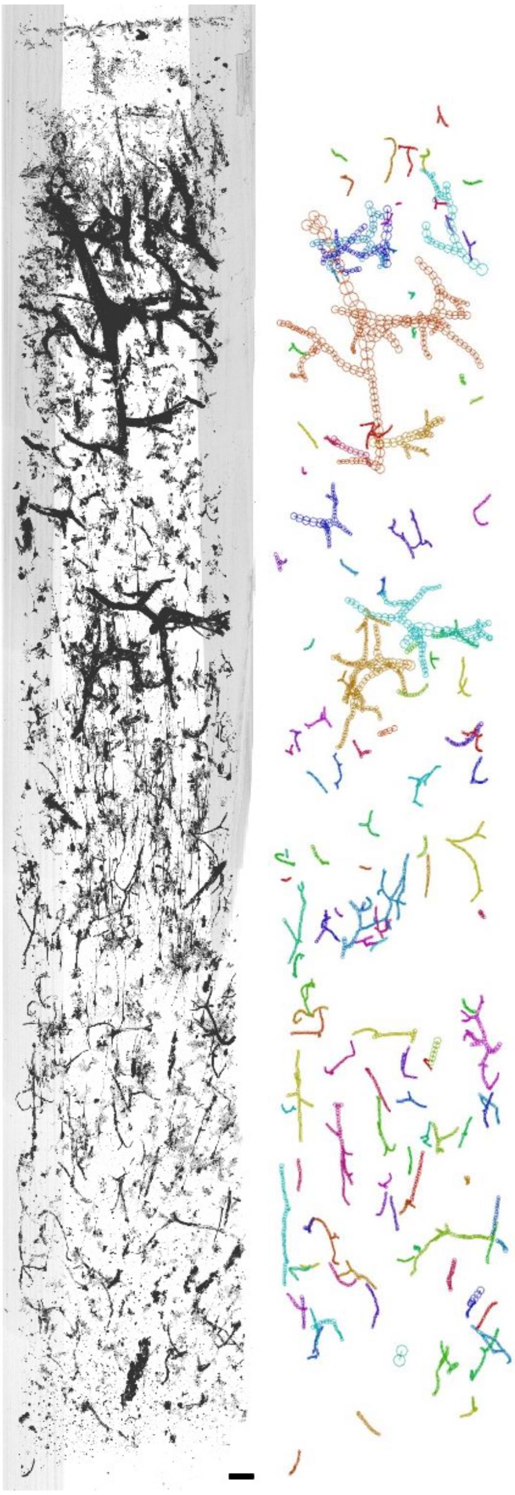

Supplementary Figure S6. Rendering of three-dimensional images of BA22 cerebral tissues and Cartesian-coordinate models of the vessel structures. Scale bars: 100 μm. (**A**) Rendering of control dataset N3-22. Linear attenuation coefficients of 8–80 cm^{-1} were rendered in gray scale. (**B**) Model of N3-22.

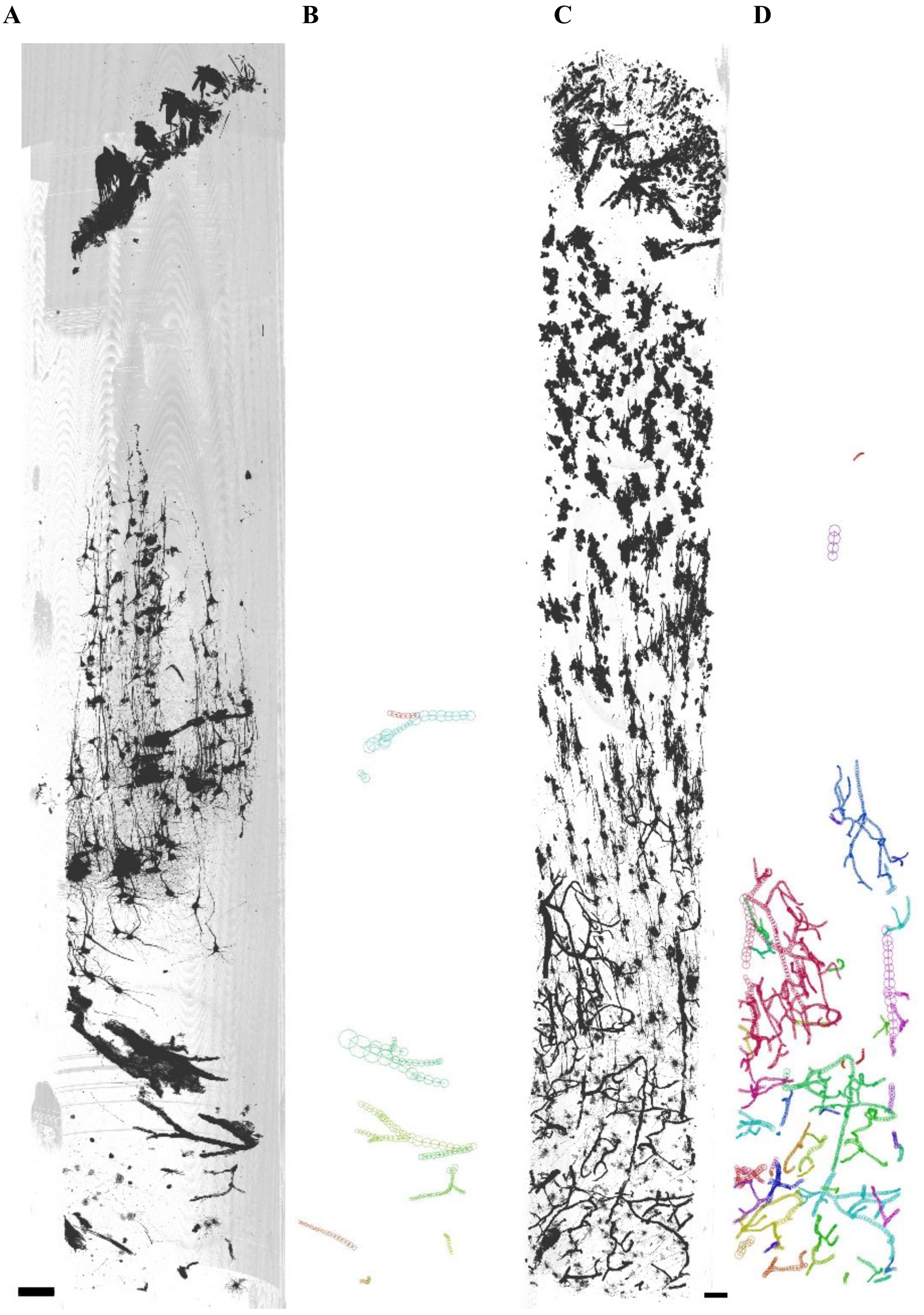

Supplementary Figure S7. Rendering of three-dimensional images of BA22 cerebral tissues and Cartesian-coordinate models of the vessel structures. Scale bars: 100 μm. (**A**) Rendering of control dataset N4-22A. Linear attenuation coefficients of 8–80 cm^{-1} were rendered in gray scale. (**B**) Model of N4-22A. (**C**) Rendering of control dataset N4-22B. Linear attenuation coefficients of 8–50 cm^{-1} were rendered in gray scale. (**D**) Model of N4-22B.

A B

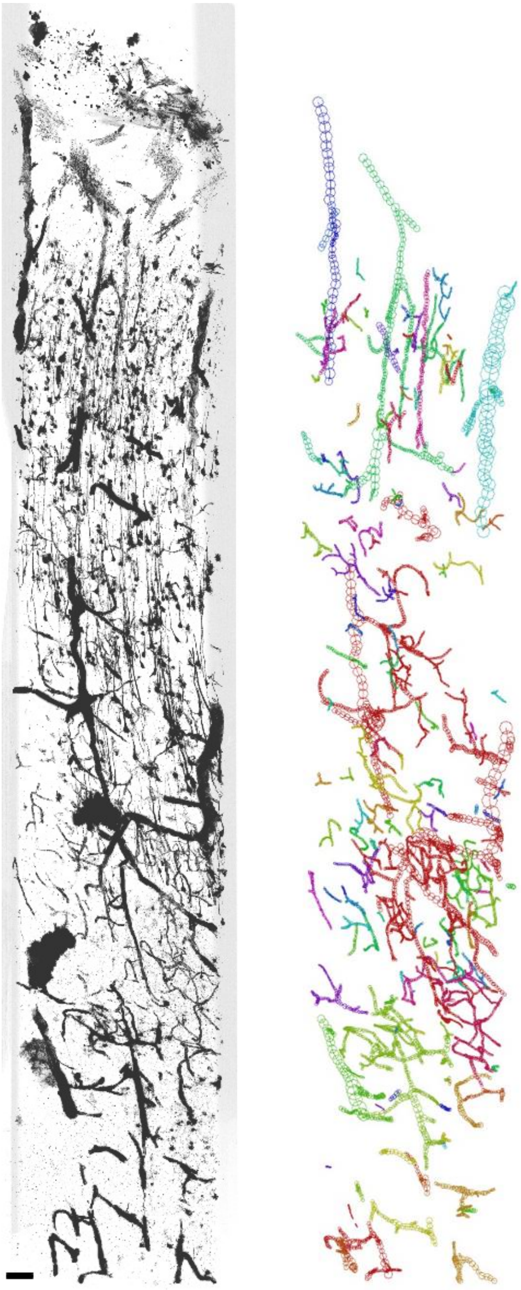

Supplementary Figure S8. Rendering of three-dimensional images of BA24 cerebral tissues and Cartesian-coordinate models of the vessel structures. Renderings and models are viewed from nearly the same direction. The pial surface is toward the top. Images were rendered with the maximum projection method of the VG Studio software. Models were drawn with the MCTrace software. Model constituents are color-coded. Nodes composing vessels are indicated with circles. Scale bars: 100 µm. (**A**) Rendering of image dataset S1-24 of the schizophrenia S1 case. Linear attenuation coefficients of 16–60 cm^{-1} were rendered in gray scale. (**B**) Cartesian-coordinate model of S1-24.

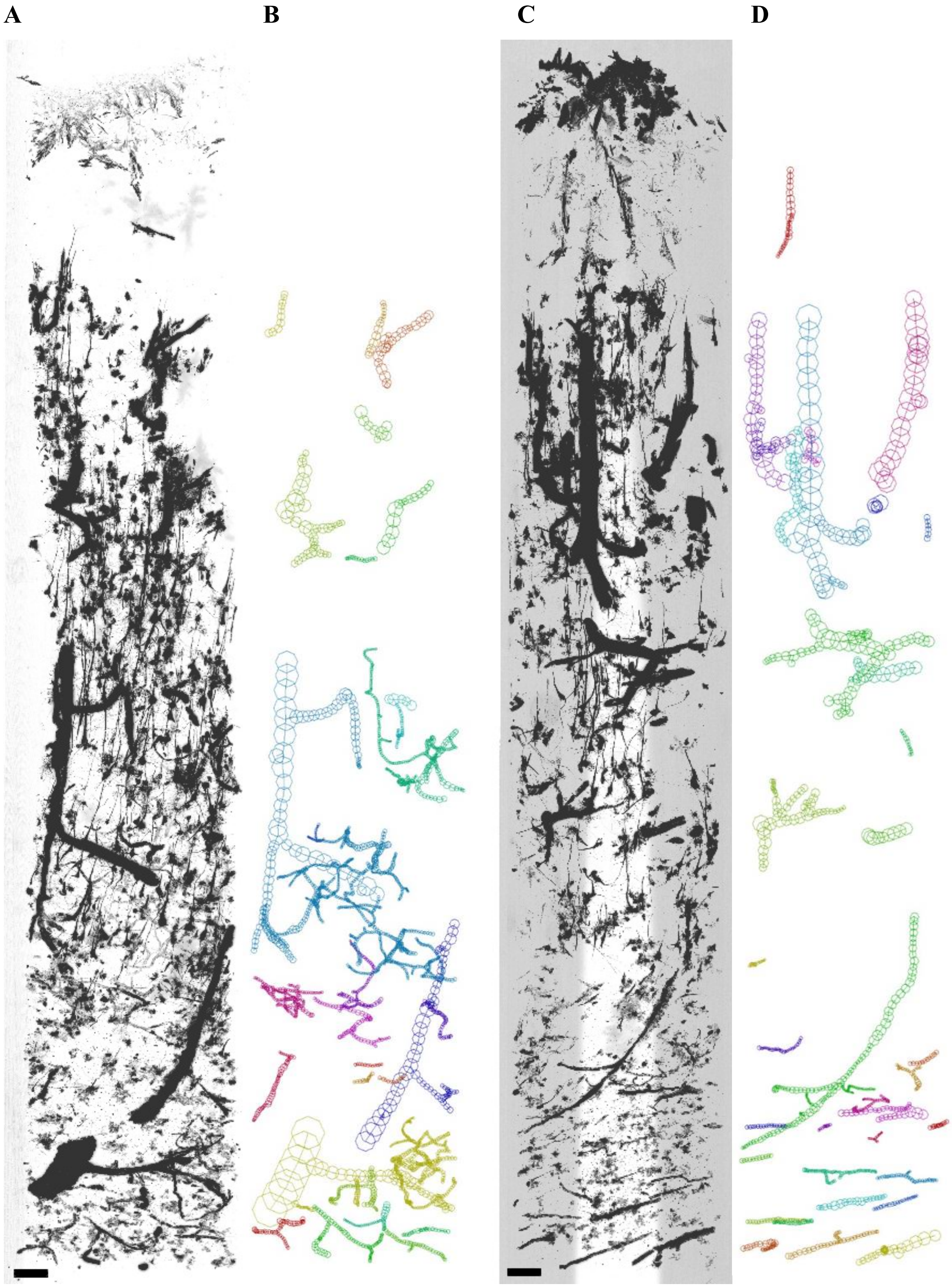

Supplementary Figure S9. Rendering of three-dimensional images of BA24 cerebral tissues and Cartesian-coordinate models of the vessel structures. Scale bars: 100 µm. (**A**) Rendering of schizophrenia dataset S2-24A. Linear attenuation coefficients of 8–50 cm^{-1} were rendered in gray scale. (**B**) Model of S2-24A. (**C**) Rendering of schizophrenia dataset S2-24B. Linear attenuation coefficients of 8–50 cm^{-1} were rendered in gray scale. (**D**) Model of S2-24B.

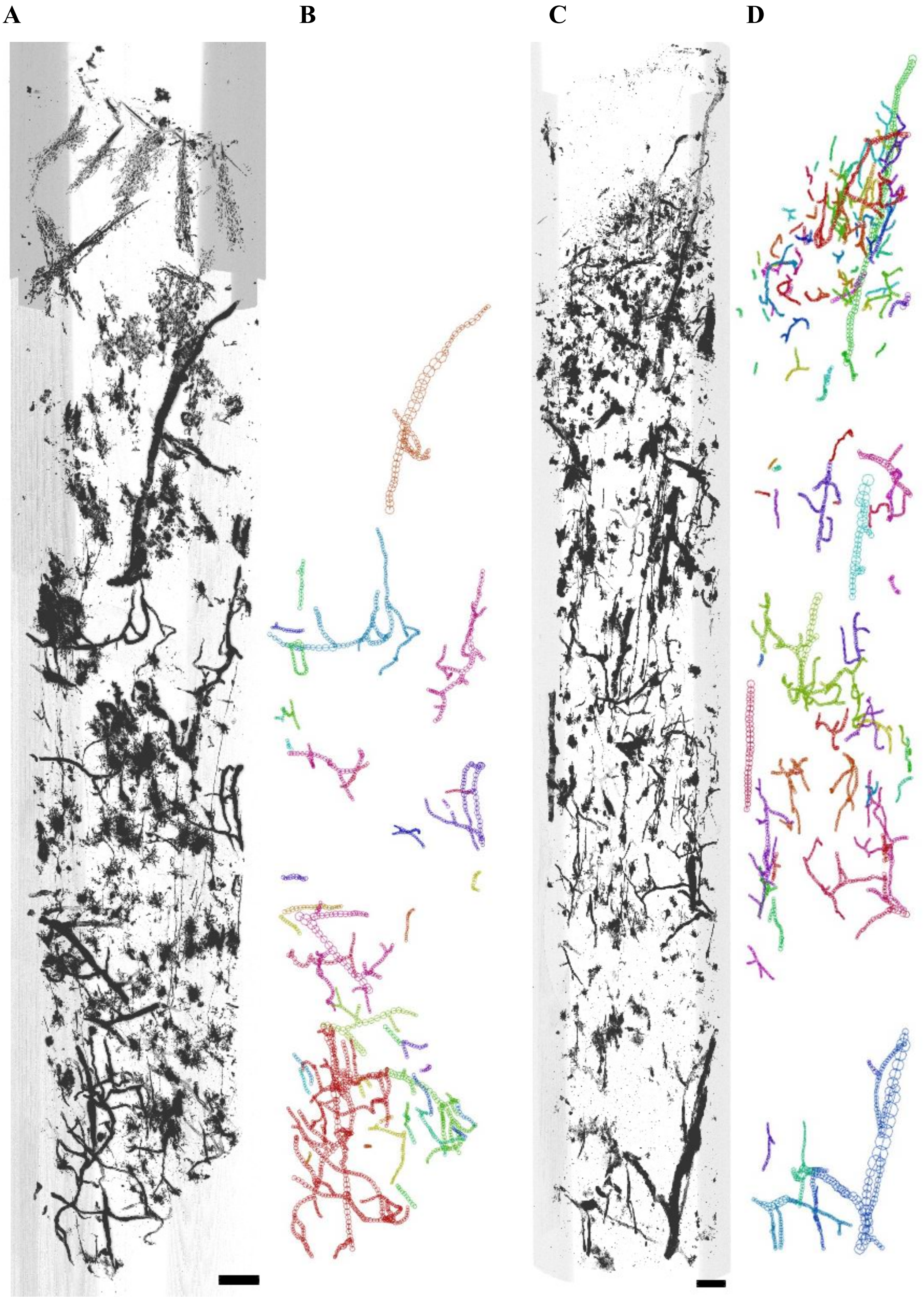

Supplementary Figure S10. Rendering of three-dimensional images of BA24 cerebral tissues and Cartesian-coordinate models of the vessel structures. Scale bars: 100 μm. (**A**) Rendering of schizophrenia dataset S3-24A. Linear attenuation coefficients of 8–50 cm^{-1} were rendered in gray scale. (**B**) Model of S3-24A. (**C**) Rendering of schizophrenia dataset S3-24B. Linear attenuation coefficients of 16–60 cm^{-1} were rendered in gray scale. (**D**) Model of S3-24B.

A B

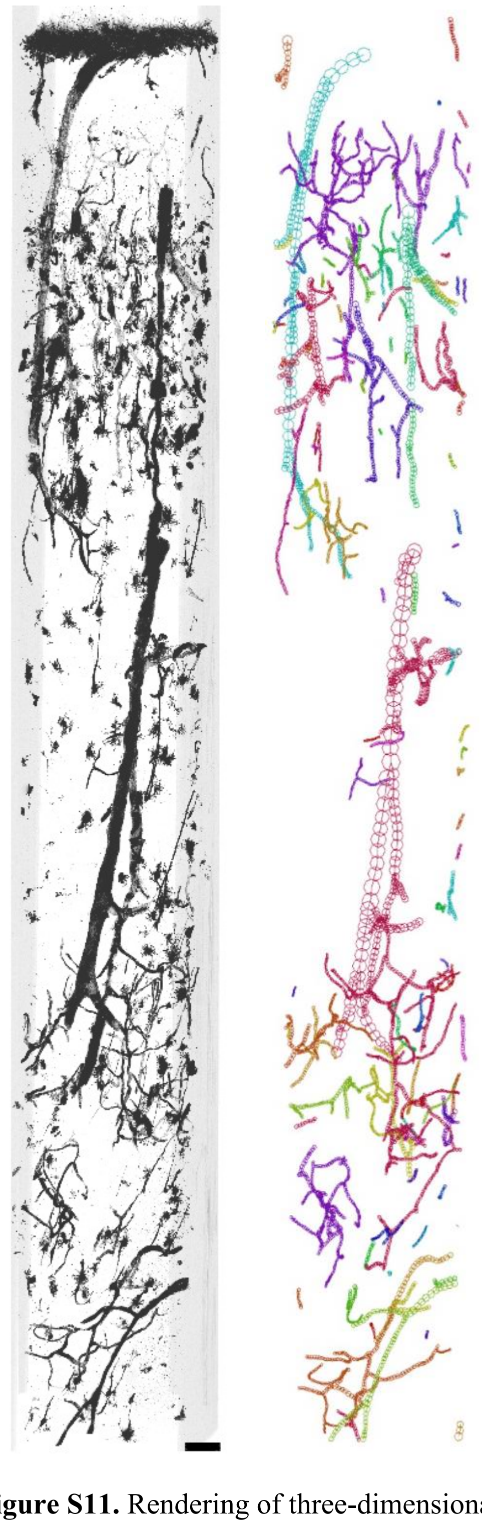

Supplementary Figure S11. Rendering of three-dimensional images of BA24 cerebral tissues and Cartesian-coordinate models of the vessel structures. Scale bars: 100 µm. (**A)** Rendering of schizophrenia dataset S3-24C. Linear attenuation coefficients of 16–60 cm^{-1} were rendered in gray scale. (**B**) Model of S3-24C.

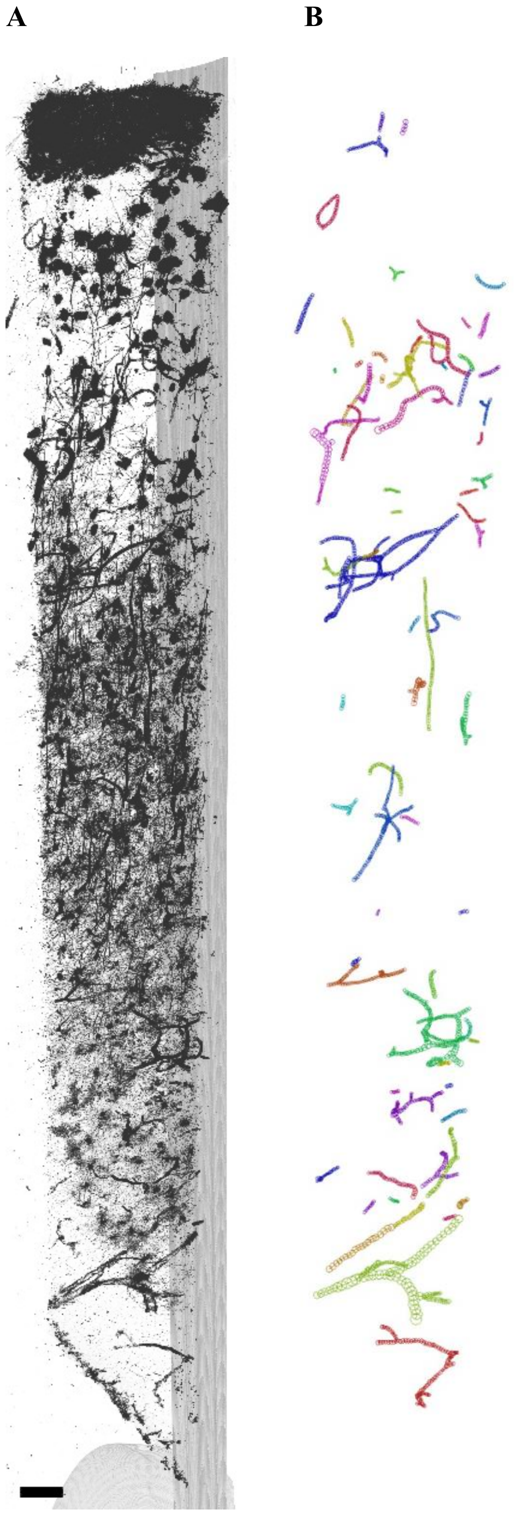

Supplementary Figure S12. Rendering of three-dimensional images of BA24 cerebral tissues and Cartesian-coordinate models of the vessel structures. Scale bars: 100 μm. (**A**) Rendering of schizophrenia dataset S4-24. Linear attenuation coefficients of 8–50 cm^{-1} were rendered in gray scale. (**B**) Model of S4-24.

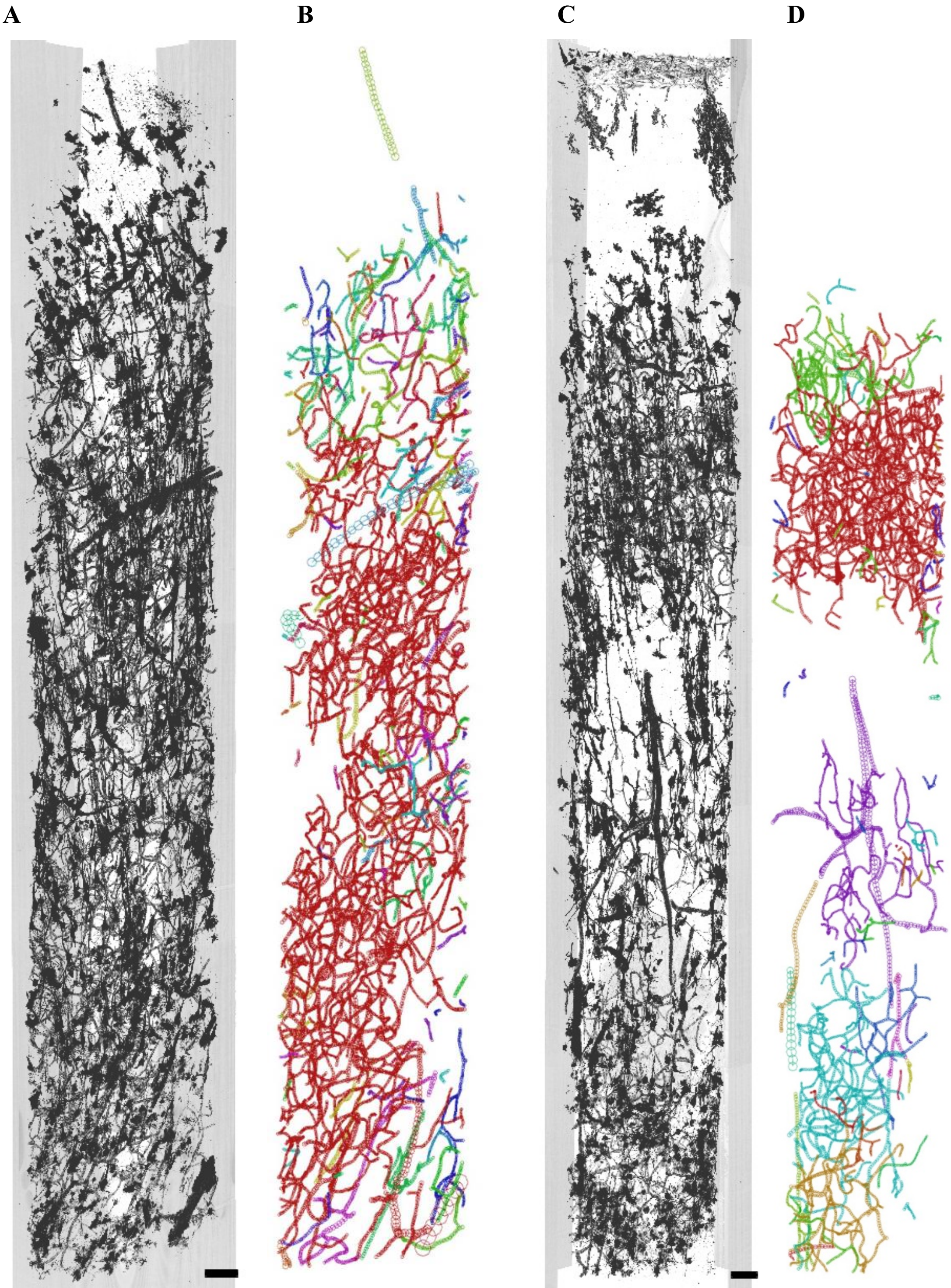

Supplementary Figure S13. Rendering of three-dimensional images of BA24 cerebral tissues and Cartesian-coordinate models of the vessel structures. Scale bars: 100 μm. (**A**) Rendering of control dataset N1-24A. Linear attenuation coefficients of 8–90 cm^{-1} were rendered in gray scale. (**B**) Model of N1-24A. (**C**) Rendering of control dataset N1-24B. Linear attenuation coefficients of 8–90 cm^{-1} were rendered in gray scale. (**D**) Model of N1-24B.

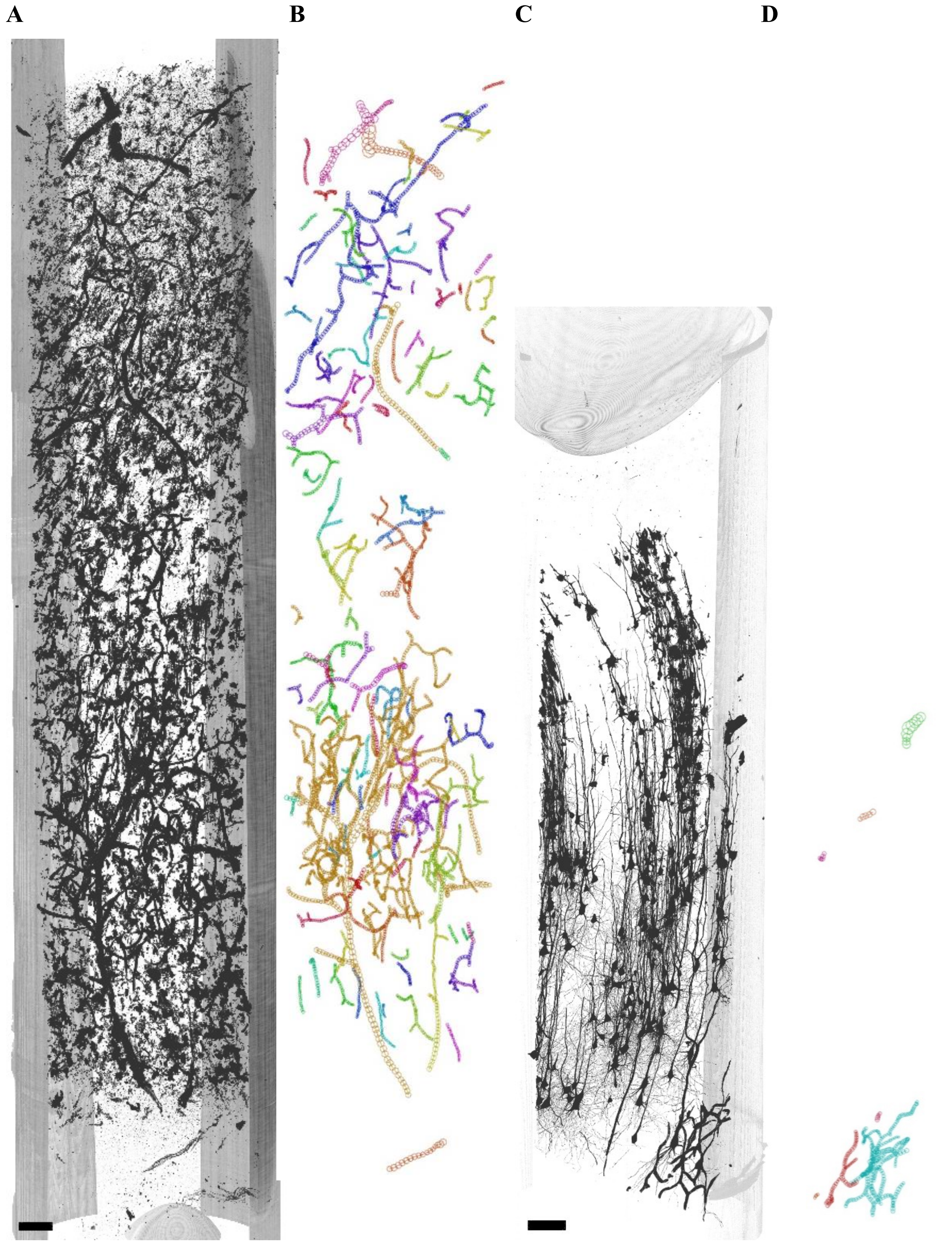

Supplementary Figure S14. Rendering of three-dimensional images of BA24 cerebral tissues and Cartesian-coordinate models of the vessel structures. Scale bars: 100 µm. (**A**) Rendering of control dataset N2-24A. Linear attenuation coefficients of 8–50 cm^{-1} were rendered in gray scale. (**B**) Model of N2-24A. (**C**) Rendering of control dataset N2-24B. Linear attenuation coefficients of 8–50 cm^{-1} were rendered in gray scale. (**D**) Model of N2-24B.

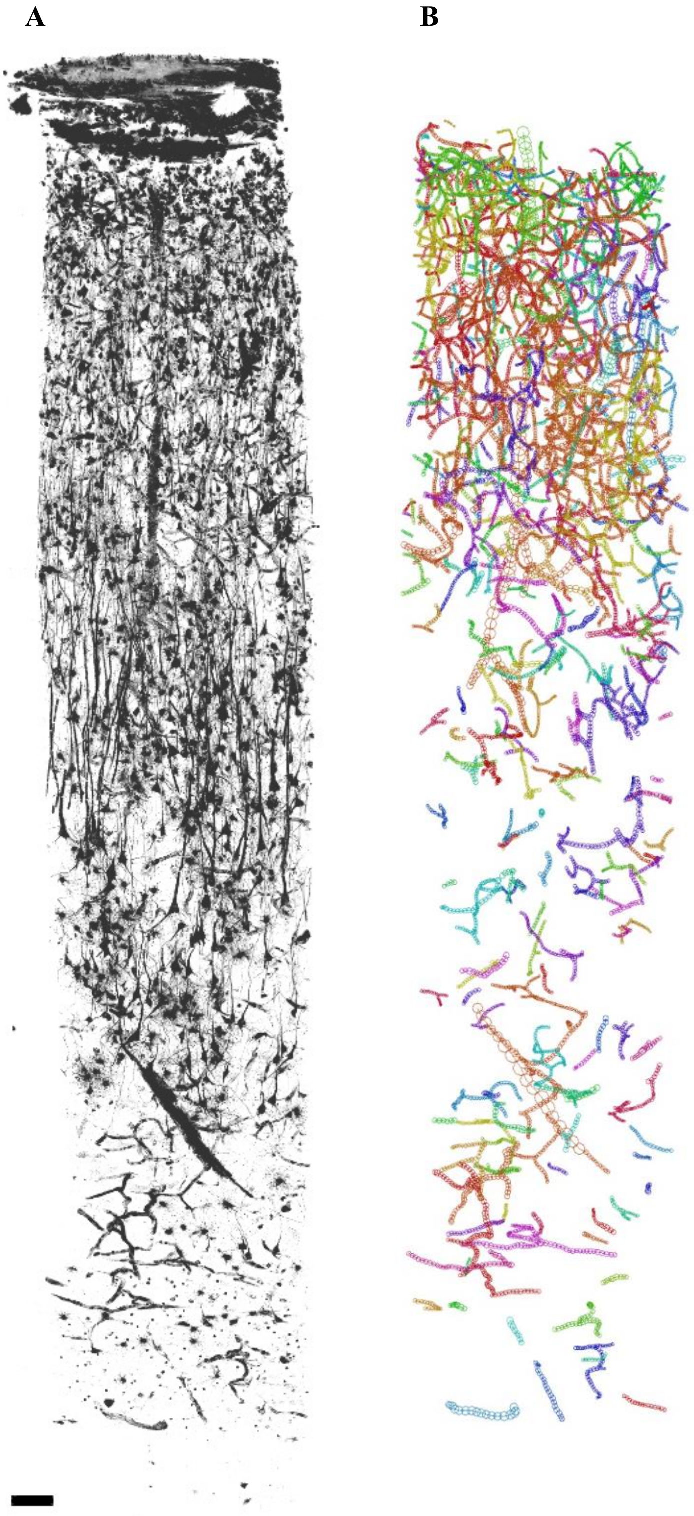

Supplementary Figure S15. Rendering of three-dimensional images of BA24 cerebral tissues and Cartesian-coordinate models of the vessel structures. Scale bars: 100 μm. (**A**) Rendering of control dataset N3-24A. Linear attenuation coefficients of 16–60 cm^{-1} were rendered in gray scale. (**B**) Model of N3-24A.

A B

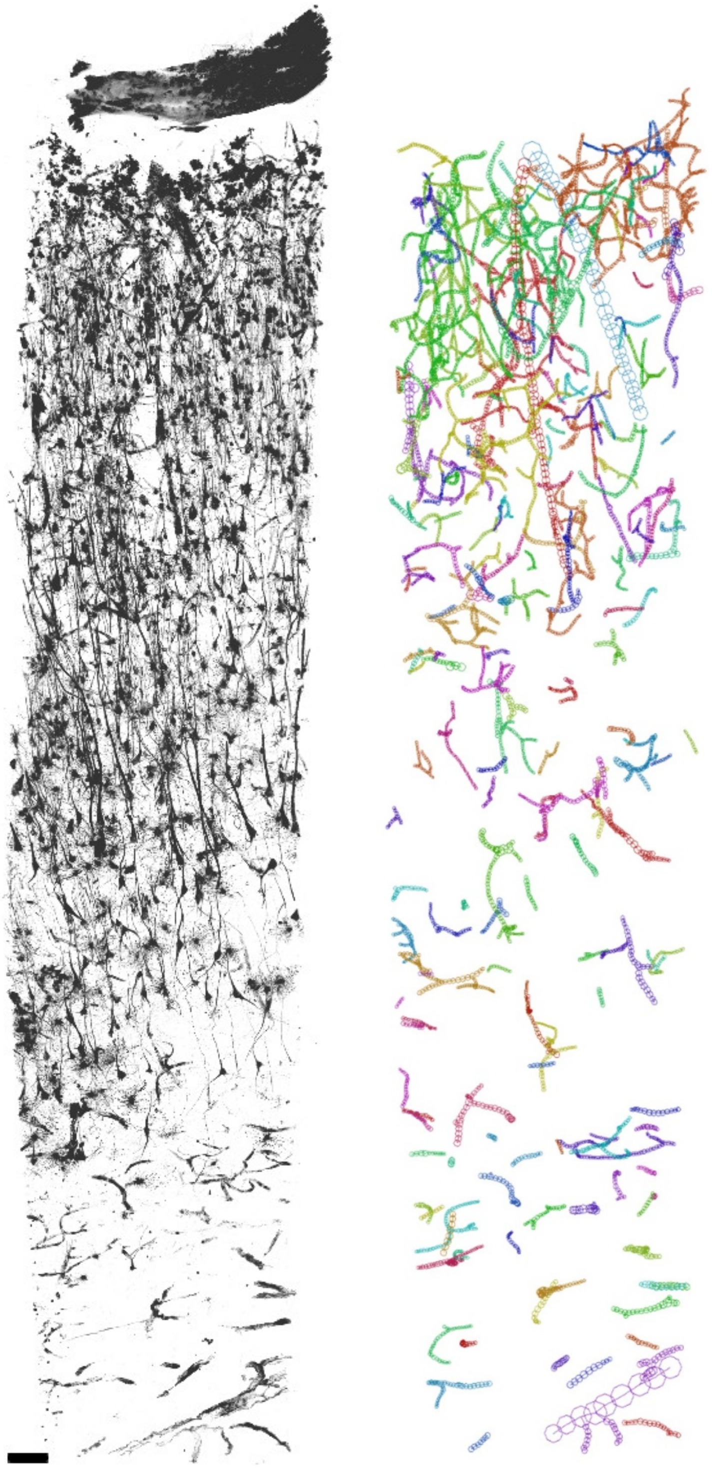

Supplementary Figure S16. Rendering of three-dimensional images of BA24 cerebral tissues and Cartesian-coordinate models of the vessel structures. Scale bars: 100 μm. (**A**) Rendering of control dataset N3-24B. Linear attenuation coefficients of 16–60 cm^{-1} were rendered in gray scale. (**B**) Model of N3-24B.

A B

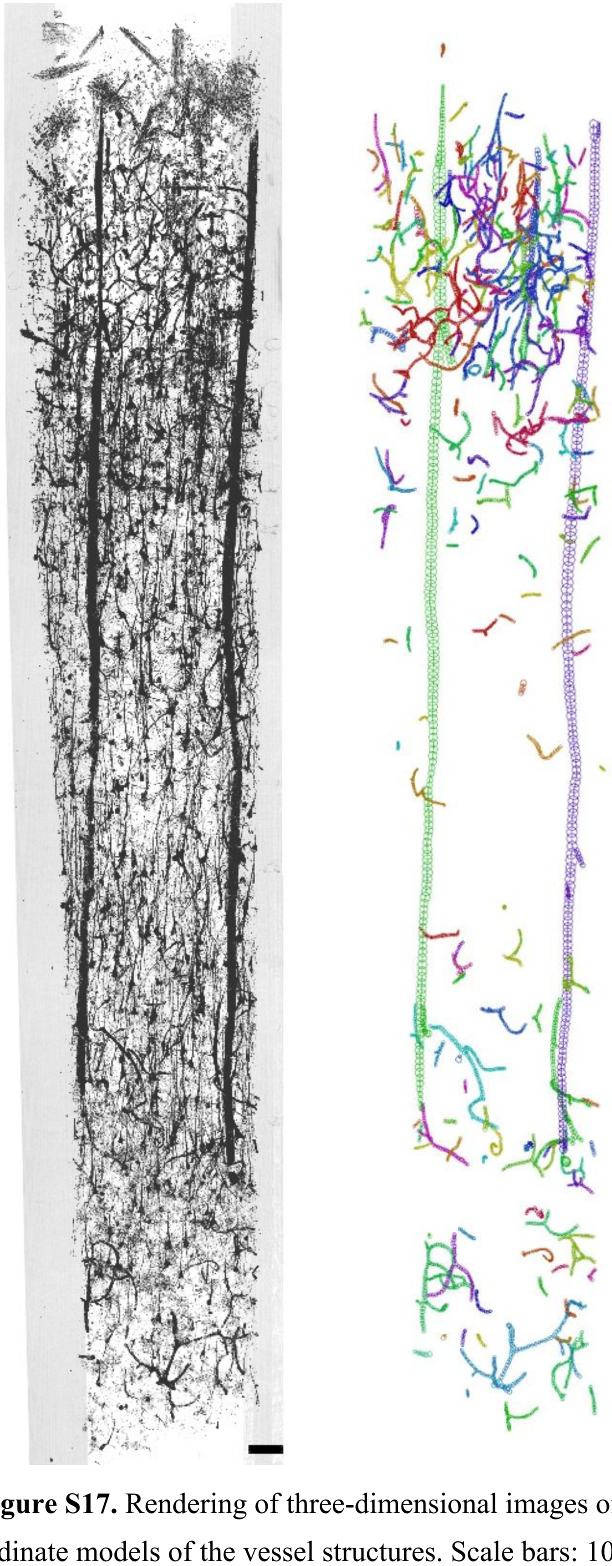

Supplementary Figure S17. Rendering of three-dimensional images of BA24 cerebral tissues and Cartesian-coordinate models of the vessel structures. Scale bars: 100 μm. (**A**) Rendering of control dataset N4-24. Linear attenuation coefficients of 8–90 cm^{-1} were rendered in gray scale. (**B**) Model of N4-24.

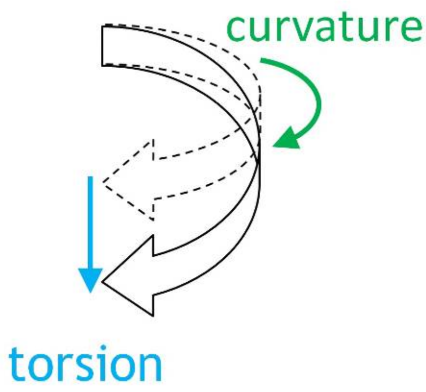

Supplementary Figure S18. Schematic drawing illustrating the definition of curvature and torsion. The curvature corresponds to the reciprocal of the radius of a curve; hence, it represents the sharpness of the capillary curve. The torsion is the deviation of the curve from a plane; it represents the right/left handedness of a spiral.

A

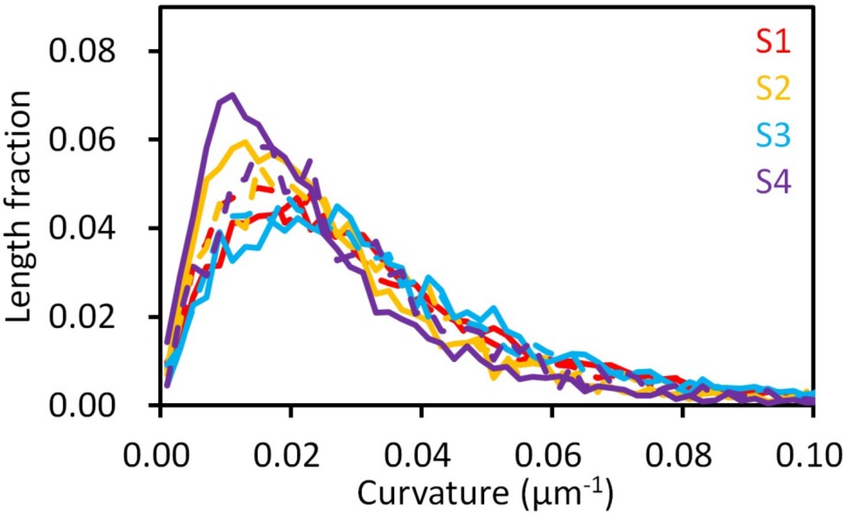

B

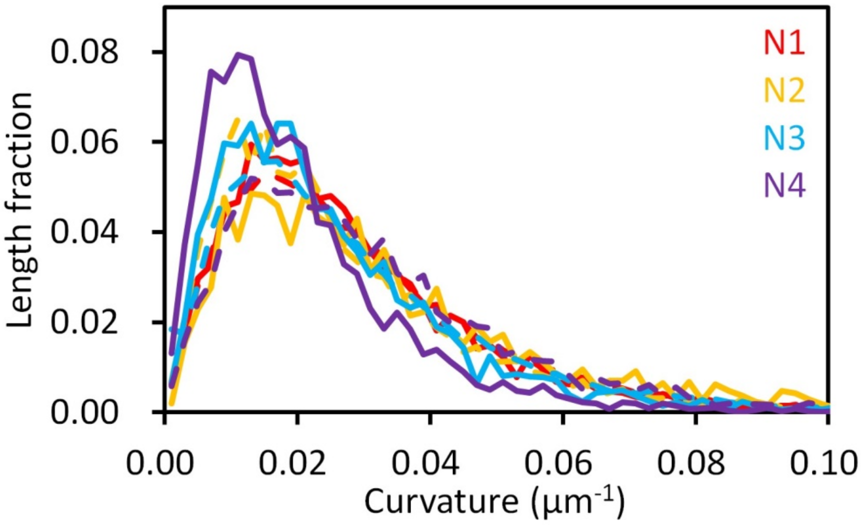

Supplementary Figure S19. Capillary curvature distribution. The frequency distribution in each 0.002-μm^{-1} curvature bin is represented by the length fraction, which was calculated by dividing the capillary length per bin by the total capillary length. Schizophrenia cases S1–S4 and controls N1–N4 are color-coded. Solid lines represent BA22 distributions, and dashed lines represent BA24 distributions. (**A**) Curvature distribution of the schizophrenia cases. (**B**) Curvature distribution of the control cases.

A

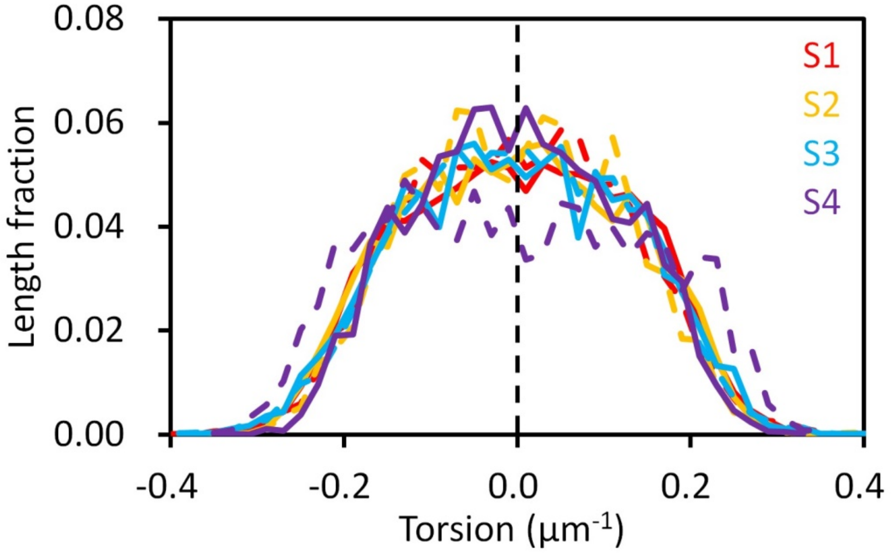

B

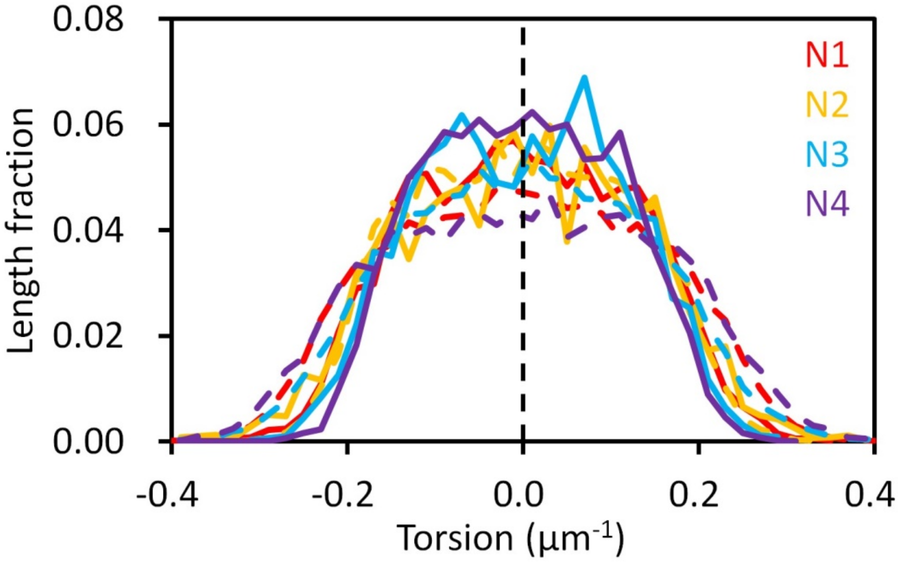

Supplementary Figure S20. Capillary torsion distribution. The frequency distribution in each 0.02-μm^{-1} torsion bin is represented by the length fraction, which was calculated by dividing the capillary length per bin by the total capillary length. Schizophrenia cases S1–S4 and controls N1–N4 are color-coded. Solid lines represent BA22 distributions, and dashed lines represent BA24 distributions. (**A**) Torsion distribution of the schizophrenia cases. (**B**) Torsion distribution of the control cases.

Supplementary Video caption

Supplementary Video S1. Structure of the schizophrenia S1-22 sample. The pial surface is toward the top. The first half shows a rendering of the three-dimensional image visualized with synchrotron radiation microtomography. Linear attenuation coefficients of 8–50 cm^{-1} were rendered in gray scale with the maximum projection method of the VG Studio software. The video crossfades to the vessel model built from the image. Model constituents are color-coded. Nodes composing vessels are indicated with octagons. Video frames of the vessel model were produced using the MCTrace software.
