# Supplementary material for: Brain capillary structures of schizophrenia cases and controls show a correlation with their neuron structures
Source: Sci Rep. 2021 Jun 3;11:11768. doi: 10.1038/s41598-021-91233-z (PMC8175464; doi:10.1038/s41598-021-91233-z)
Supplement: Supplementary file 2 — Supplementary Information 2. [file 41598_2021_91233_MOESM2_ESM.pdf]

Supplementary Tables of:

Brain capillary structures of schizophrenia cases and controls show a correlation with their neuron structures

Rino Saiga[1], Masayuki Uesugi[2], Akihisa Takeuchi[2], Kentaro Uesugi[2], Yoshio Suzuki[3], Susumu Takekoshi[4], Chie Inomoto[5], Naoya Nakamura[5], Youta Torii[6], Itaru Kushima[6,7], Shuji Iritani[6,8], Norio Ozaki[6,7], Kenichi Oshima[8,9], Masanari Itokawa[8,9], Makoto Arai[9], and Ryuta Mizutani[1,*]

[1]Department of Applied Biochemistry, Tokai University, Hiratsuka, Kanagawa 259-1292, Japan;
[2]Japan Synchrotron Radiation Research Institute (JASRI/SPring-8), Sayo, Hyogo 679-5198, Japan;
[3]Photon Factory, High Energy Accelerator Research Organization KEK, Tsukuba, Ibaraki 305-0801, Japan;
[4]Department of Cell Biology, Tokai University School of Medicine, Isehara, Kanagawa 259-1193, Japan;
[5]Department of Pathology, Tokai University School of Medicine, Isehara, Kanagawa 259-1193, Japan;
[6]Department of Psychiatry, Nagoya University Graduate School of Medicine, Nagoya, Aichi 466-8550, Japan;
[7]Medical Genomics Center, Nagoya University Hospital, Nagoya 466-8550, Aichi, Japan;
[8]Tokyo Metropolitan Matsuzawa Hospital, Setagaya, Tokyo 156-0057, Japan;
[9]Tokyo Metropolitan Institute of Medical Science, Setagaya, Tokyo 156-8506, Japan
*Correspondence: mizutanilaboratory@gmail.com

Supplementary table S1. Statistics of structural analysis.

Case code	S1	S2	S3	S4	N1	N2	N3	N4
Gender	female	female	male	male	female	female	male	male
Age	56	70	64	69	58	72	62	65
Postmortem interval (hour)	2	7.5	21	7	85	3.5	8	17.5
Psychiatric record	schizophrenia	schizophrenia	schizophrenia	schizophrenia	no	no	no	no
BA22 structure								
Number of datasets	1	1	1	1	2	1	1	2
Number of model nodes	11326	6437	4274	4574	7152	2473	4153	5105
Total vessel length (um)	73730.0	44264.1	29994.9	31154.0	49069.8	16966.5	40036.2	45089.9
Overall vessel								
Diameter (um) [a]	8.6 (0.03)	9.7 (0.12)	10.6 (0.15)	8.5 (0.03)	9.8 (0.12)	10.6 (0.26)	16.7 (0.30)	13.2 (0.14)
Curvature (um^{-1}) [a]	0.0351 (0.0002)	0.0258 (0.0003)	0.0342 (0.0004)	0.0243 (0.0003)	0.0274 (0.0002)	0.0355 (0.0006)	0.0212 (0.0003)	0.0191 (0.0002)
Torsion (um^{-1}) [a]	0.0021 (0.0011)	-0.0015 (0.0013)	-0.0005 (0.0016)	0.0000 (0.0015)	-0.0004 (0.0013)	0.0001 (0.0022)	-0.0009 (0.0012)	-0.0011 (0.0012)
Capillary								
Diameter (um) [a]	8.2 (0.02)	8.3 (0.03)	8.6 (0.05)	8.4 (0.03)	8.9 (0.02)	8.6 (0.05)	9.7 (0.05)	10.6 (0.04)
Curvature (um^{-1}) [a]	0.0358 (0.0003)	0.0271 (0.0003)	0.0371 (0.0004)	0.0245 (0.0003)	0.0284 (0.0002)	0.0378 (0.0007)	0.0259 (0.0003)	0.0209 (0.0002)
Torsion (um^{-1}) [a]	0.0021 (0.0011)	-0.0018 (0.0014)	-0.0002 (0.0018)	0.0001 (0.0016)	-0.0007 (0.0013)	-0.0003 (0.0024)	-0.0014 (0.0017)	-0.0013 (0.0014)
Neurite curvature (um^{-1}) [a,b]	1.08 (0.01)	0.56 (0.01)	0.63 (0.01)	0.36 (0.01)	0.28 (0.01)	0.58 (0.01)	0.46 (0.01)	0.35 (0.01)
Neurite radius (um) [a,b]	0.24 (0.01)	0.44 (0.01)	0.38 (0.01)	0.71 (0.02)	1.16 (0.05)	0.45 (0.02)	0.48 (0.02)	0.61 (0.03)
BA24 structure								
Number of datasets	1	2	3	1	2	2	2	1
Number of model nodes	8700	3567	12865	2195	39342	6839	26009	7913
Total vessel length (um)	70307.2	36065.7	95782.6	12960.7	231412.5	48199.6	164998.5	48788.8
Overall vessel								
Diameter (um) [a]	13.6 (0.17)	22.0 (0.56)	11.8 (0.11)	9.9 (0.12)	8.7 (0.03)	9.9 (0.06)	9.5 (0.05)	11.1 (0.13)
Curvature (um^{-1}) [a]	0.0291 (0.0003)	0.0230 (0.0004)	0.0314 (0.0002)	0.0294 (0.0005)	0.0296 (0.0001)	0.0262 (0.0002)	0.0281 (0.0001)	0.0277 (0.0003)
Torsion (um^{-1}) [a]	-0.0007 (0.0010)	-0.0022 (0.0013)	0.0001 (0.0009)	-0.0027 (0.0027)	-0.0008 (0.0006)	-0.0012 (0.0012)	-0.0005 (0.0007)	0.0022 (0.0014)
Capillary								
Diameter (um) [a]	9.2 (0.04)	9.9 (0.07)	9.2 (0.03)	9.0 (0.06)	8.3 (0.01)	9.1 (0.03)	8.5 (0.02)	8.4 (0.03)
Curvature (um^{-1}) [a]	0.0336 (0.0003)	0.0316 (0.0005)	0.0349 (0.0002)	0.0307 (0.0005)	0.0302 (0.0001)	0.0273 (0.0003)	0.0291 (0.0001)	0.0313 (0.0003)
Torsion (um^{-1}) [a]	-0.0010 (0.0012)	-0.0033 (0.0020)	-0.0001 (0.0010)	-0.0021 (0.0029)	-0.0009 (0.0007)	-0.0007 (0.0013)	-0.0003 (0.0008)	0.0023 (0.0016)
Neurite curvature (um^{-1}) [a,c]	0.46 (0.01)	0.47 (0.01)	0.60 (0.02)	0.71 (0.01)	0.33 (0.01)	0.44 (0.01)	0.37 (0.01)	0.41 (0.01)
Neurite radius (um) [a,c]	0.66 (0.03)	0.63 (0.02)	0.58 (0.03)	0.39 (0.01)	1.06 (0.05)	0.53 (0.01)	0.71 (0.03)	0.59 (0.02)

[a] Mean (standard error)

[b] Transl Psychiatry 11: 49 (2021)

[c] Transl Psychiatry 9: 85 (2019)

Supplementary table S2. Conditions of microtomography experiments.

Beamtime start date	2011.12.8	2013.1.23	2013.6.8	2013.12.11	2015.6.2	2015.6.22	2015.11.5	2016.9.23	2018.5.15
Scintillator screen	LSO (Lu_2SiO_5:Ce)	LSO	LSO	LuAG:Ce ($Lu_3Al_5O_{12}$:Ce)	LuAG:Ce	LuAG:Ce	LuAG:Ce	LuAG:Ce	GAGG:Ce ($Gd_3Al_2Ga_3O_{12}$:Ce)
Camera	ORCA-Flash2.8	ORCA-Flash4.0	ORCA-Flash4.0	ORCA-Flash4.0	ORCA-Flash4.0	ORCA-Flash4.0	ORCA-Flash4.0	ORCA-Flash4.0 V2	ORCA-Flash4.0 V3
Pixel size (μm)	0.500	0.504	0.505	0.508	0.504	0.504	0.500	0.510	0.510
Viewing field (pixels) [a]	1920 x 1440	2048 x 2048	2048 x 2048	2048 x 2048	2048 x 2048	2048 x 2048	2048 x 2048	2048 x 2048	2048 x 2048
Image dynamic range (bits) [b]	12	15	16	16	16	16	15	15	15
Number of sample frames per dataset	1800	1800	1800	1800	1800	1800	1800	1800	1800
Degrees per frame	0.100	0.100	0.100	0.100	0.100	0.100	0.100	0.100	0.100
Exposure time per frame (msec)	200	150	150	150	150	150	100	150	150
Data collection time (sec)	1500	600	600	600	550	550	400	450	450

[a] Width x height

[b] Defined from the maximum intensity of flat field images

Supplementary table S3. Statistics of datasets and Cartesian coordinate models. (**A**) Schizophrenia case S1.

Dataset name	S1-22	S1-24
Brain area	BA22	BA24
Beamtime start date	2016.9.23	2013.1.23
Image size (pixel) [a]	1380 x 1220 x 5481	1280 x 1620 x 8900
Image size (um) [a]	704 x 622 x 2795	645 x 816 x 4486
Overall vessel		
Number of nodes	11326	8700
Total length (um)	73730.0	70307.2
Diameter (um) [b]	8.6 (0.03)	13.6 (0.17)
Curvature (um^{-1}) [b]	0.0351 (0.0002)	0.0291 (0.0003)
Torsion (um^{-1}) [b]	0.0021 (0.0011)	-0.0007 (0.0010)
Capillary		
Total length (um)	71090.8	52569.3
Diameter (um) [b]	8.2 (0.02)	9.2 (0.04)
Curvature (um^{-1}) [b]	0.0358 (0.0003)	0.0336 (0.0003)
Torsion (um^{-1}) [b]	0.0021 (0.0011)	-0.0010 (0.0012)

[a] Image width x height x number of slices.

[b] Mean (standard error)

Supplementary table S3. Statistics of datasets and Cartesian coordinate models. (**B**) Schizophrenia case S2.

Dataset name	S2-22	S2-24A	S2-24B
Brain area	BA22	BA24	BA24
Beamtime start date	2013.12.11	2013.6.8	2013.6.8
Image size (pixel) [a]	1220 x 1450 x 7367	1360 x 1440 x 7394	1310 x 1430 x 7397
Image size (um) [a]	620 x 737 x 3742	687 x 727 x 3734	662 x 722 x 3735
Overall vessel			
Number of nodes	6437	2609	958
Total length (um)	44264.1	23726.1	12339.6
Diameter (um) [b]	9.7 (0.12)	18.5 (0.74)	28.9 (0.83)
Curvature (um^{-1}) [b]	0.0258 (0.0003)	0.0259 (0.0005)	0.0173 (0.0005)
Torsion (um^{-1}) [b]	-0.0015 (0.0013)	-0.0013 (0.0016)	-0.0040 (0.0019)
Capillary			
Total length (um)	40277.7	15708.6	3924.7
Diameter (um) [b]	8.3 (0.03)	9.8 (0.07)	10.6 (0.16)
Curvature (um^{-1}) [b]	0.0271 (0.0003)	0.0324 (0.0005)	0.0283 (0.0010)
Torsion (um^{-1}) [b]	-0.0018 (0.0014)	-0.0017 (0.0022)	-0.0095 (0.0044)

[a] Image width x height x number of slices.

[b] Mean (standard error)

Supplementary table S3. Statistics of datasets and Cartesian coordinate models. (**C**) Schizophrenia case S3.

Dataset name	S3-22	S3-24A	S3-24B	S3-24C
Brain area	BA22	BA24	BA24	BA24
Beamtime start date	2018.5.14	2013.1.23	2013.1.23	2013.1.23
Image size (pixel) [a]	1590 x 1310 x 7346	1190 x 1090 x 6806	1140 x 1280 x 7951	980 x 1140 x 7950
Image size (um) [a]	811 x 668 x 3746	600 x 549 x 3430	575 x 645 x 4007	494 x 575 x 4007
Overall vessel				
Number of nodes	4274	2227	4753	5885
Total length (um)	29994.9	17498.2	33804.9	44479.5
Diameter (um) [b]	10.6 (0.15)	10.5 (0.11)	11.8 (0.18)	12.2 (0.19)
Curvature (um^{-1}) [b]	0.0342 (0.0004)	0.0317 (0.0005)	0.0328 (0.0004)	0.0303 (0.0004)
Torsion (um^{-1}) [b]	-0.0005 (0.0016)	0.0032 (0.0020)	-0.0014 (0.0015)	0.0001 (0.0013)
Capillary				
Total length (um)	25932.5	16039.2	27844.1	36132.1
Diameter (um) [b]	8.6 (0.05)	9.7 (0.07)	9.1 (0.04)	9.1 (0.04)
Curvature (um^{-1}) [b]	0.0371 (0.0004)	0.0330 (0.0005)	0.0367 (0.0004)	0.0343 (0.0004)
Torsion (um^{-1}) [b]	-0.0002 (0.0018)	0.0031 (0.0021)	-0.0020 (0.0017)	0.0001 (0.0015)

[a] Image width x height x number of slices.

[b] Mean (standard error)

Supplementary table S3. Statistics of datasets and Cartesian coordinate models. (**D**) Schizophrenia case S4.

Dataset name	S4-22	S4-24
Brain area	BA22	BA24
Beamtime start date	2013.12.11	2013.12.11
Image size (pixel) [a]	1250 x 1060 x 7352	1150 x 1090 x 7352
Image size (um) [a]	635 x 538 x 3735	584 x 554 x 3735
Overall vessel		
Number of nodes	4574	2195
Total length (um)	31154.0	12960.7
Diameter (um) [b]	8.5 (0.03)	9.9 (0.12)
Curvature (um^{-1}) [b]	0.0243 (0.0003)	0.0294 (0.0005)
Torsion (um^{-1}) [b]	0.0000 (0.0015)	-0.0027 (0.0027)
Capillary		
Total length (um)	30760.9	11926.0
Diameter (um) [b]	8.4 (0.03)	9.0 (0.06)
Curvature (um^{-1}) [b]	0.0245 (0.0003)	0.0307 (0.0005)
Torsion (um^{-1}) [b]	0.0001 (0.0016)	-0.0021 (0.0029)

[a] Image width x height x number of slices.

[b] Mean (standard error)

Supplementary table S3. Statistics of datasets and Cartesian coordinate models. (**E**) Control case N1.

Dataset name	N1-22A	N1-22B	N1-24A	N1-24B
Brain area	BA22	BA22	BA24	BA24
Beamtime start date	2015.6.2	2015.6.2	2015.6.2	2015.6.2
Image size (pixel) [a]	1210 x 1180 x 7406	1000 x 1280 x 7405	1310 x 1260 x 7406	1360 x 1420 x 9192
Image size (um) [a]	610 x 595 x 3733	504 x 645 x 3732	660 x 635 x 3733	685 x 716 x 4633
Overall vessel				
Number of nodes	995	6157	22683	16659
Total length (um)	7794.6	41275.2	126086.9	105325.6
Diameter (um) [b]	10.2 (0.10)	9.7 (0.14)	8.6 (0.04)	9.0 (0.04)
Curvature (um^{-1}) [b]	0.0243 (0.0005)	0.0280 (0.0003)	0.0301 (0.0001)	0.0290 (0.0002)
Torsion (um^{-1}) [b]	-0.0052 (0.0030)	0.0005 (0.0014)	-0.0027 (0.0009)	0.0016 (0.0009)
Capillary				
Total length (um)	7120.6	39558.7	123247.6	100970.7
Diameter (um) [b]	9.6 (0.07)	8.7 (0.02)	8.2 (0.01)	8.4 (0.02)
Curvature (um^{-1}) [b]	0.0254 (0.0006)	0.0289 (0.0003)	0.0305 (0.0001)	0.0299 (0.0002)
Torsion (um^{-1}) [b]	-0.0052 (0.0032)	0.0001 (0.0014)	-0.0028 (0.0009)	0.0015 (0.0009)

[a] Image width x height x number of slices.

[b] Mean (standard error)

Supplementary table S3. Statistics of datasets and Cartesian coordinate models. (**F**) Control case N2.

Dataset name	N2-22	N2-24A	N2-24B
Brain area	BA22	BA24	BA24
Beamtime start date	2015.11.5	2015.6.22	2015.11.5
Image size (pixel) [a]	1470 x 1400 x 5647	1500 x 1200 x 7400	1360 x 1200 x 4747
Image size (um) [a]	735 x 700 x 2824	756 x 605 x 3730	680 x 600 x 2374
Overall vessel			
Number of nodes	2473	6428	411
Total length (um)	16966.5	45408.4	2791.2
Diameter (um) [b]	10.6 (0.26)	9.9 (0.06)	9.9 (0.34)
Curvature (um^{-1}) [b]	0.0355 (0.0006)	0.0261 (0.0003)	0.0275 (0.0011)
Torsion (um^{-1}) [b]	0.0001 (0.0022)	-0.0003 (0.0013)	-0.0145 (0.0052)
Capillary			
Total length (um)	15094.9	41902.1	2652.5
Diameter (um) [b]	8.6 (0.05)	9.1 (0.03)	9.0 (0.10)
Curvature (um^{-1}) [b]	0.0378 (0.0007)	0.0273 (0.0003)	0.0284 (0.0011)
Torsion (um^{-1}) [b]	-0.0003 (0.0024)	0.0003 (0.0014)	-0.0157 (0.0054)

[a] Image width x height x number of slices.

[b] Mean (standard error)

Supplementary table S3. Statistics of datasets and Cartesian coordinate models. (**G**) Control case N3.

Dataset name	N3-22	N3-24A	N3-24B
Brain area	BA22	BA24	BA24
Beamtime start date	2018.5.14	2011.12.8	2011.12.8
Image size (pixel) [a]	1620 x 1980 x 12649	1410 x 1520 x 7048	1190 x 1630 x 7038
Image size (um) [a]	826 x 1010 x 6451	705 x 760 x 3524	595 x 815 x 3519
Overall vessel			
Number of nodes	4153	16831	9178
Total length (um)	40036.2	102392.1	62606.4
Diameter (um) [b]	16.7 (0.30)	9.1 (0.05)	10.0 (0.12)
Curvature (um^{-1}) [b]	0.0212 (0.0003)	0.0283 (0.0002)	0.0279 (0.0002)
Torsion (um^{-1}) [b]	-0.0009 (0.0012)	-0.0002 (0.0009)	-0.0008 (0.0011)
Capillary			
Total length (um)	25830.0	97318.7	57247.8
Diameter (um) [b]	9.7 (0.05)	8.5 (0.02)	8.7 (0.03)
Curvature (um^{-1}) [b]	0.0259 (0.0003)	0.0290 (0.0002)	0.0292 (0.0002)
Torsion (um^{-1}) [b]	-0.0014 (0.0017)	-0.0003 (0.0010)	-0.0004 (0.0012)

[a] Image width x height x number of slices.

[b] Mean (standard error)

Supplementary table S3. Statistics of datasets and Cartesian coordinate models. (**H**) Control case N4.

Dataset name	N4-22A	N4-22B	N4-24
Brain area	BA22	BA22	BA24
Beamtime start date	2018.5.14	2016.9.23	2015.6.22
Image size (pixel) [a]	1370 x 1380 x 7348	1720 x 1400 x 10335	1340 x 1520 x 9186
Image size (um) [a]	699 x 704 x 3747	877 x 714 x 5271	675 x 766 x 4630
Overall vessel			
Number of nodes	199	4906	7913
Total length (um)	2647.5	42442.5	48788.8
Diameter (um) [b]	21.3 (1.02)	12.7 (0.14)	11.1 (0.13)
Curvature (um^{-1}) [b]	0.0134 (0.0008)	0.0194 (0.0002)	0.0277 (0.0003)
Torsion (um^{-1}) [b]	0.0111 (0.0041)	-0.0018 (0.0013)	0.0022 (0.0014)
Capillary			
Total length (um)	793.2	33845.9	41577.8
Diameter (um) [b]	10.3 (0.27)	10.6 (0.04)	8.4 (0.03)
Curvature (um^{-1}) [b]	0.0168 (0.0014)	0.0210 (0.0002)	0.0313 (0.0003)
Torsion (um^{-1}) [b]	0.0172 (0.0081)	-0.0017 (0.0015)	0.0023 (0.0016)

[a] Image width x height x number of slices.

[b] Mean (standard error)
